# S100A9-Driven Amyloid-Neuroinflammatory Cascade in Traumatic Brain Injury as a Precursor State for Alzheimer’s Disease

**DOI:** 10.1038/s41598-018-31141-x

**Published:** 2018-08-27

**Authors:** Chao Wang, Igor A. Iashchishyn, Jonathan Pansieri, Sofie Nyström, Oxana Klementieva, John Kara, Istvan Horvath, Roman Moskalenko, Reza Rofougaran, Gunnar Gouras, Gabor G. Kovacs, S. K. Shankar, Ludmilla A. Morozova-Roche

**Affiliations:** 1 Department of Medical Biochemistry and Biophysics, Umeå University, 90187 Umeå, Sweden; 2 Department of General Chemistry, Sumy State University, Sumy, 40000 Ukraine; 3 IFM-Department of Chemistry, Linköping University, 58183, Linköping, Sweden; 4 Department of Experimental Medical Sciences, Lund University, 22184, Lund, Sweden; 5 Department of Pathology, Sumy State University, Sumy, 40000 Ukraine; 6 Institute of Neurology, Medical University of Vienna, 1097, Vienna, Austria; 7 Human Brain Tissue Repository, Department of Neuropathology, National Institute of Mental Health and Neurosciences, 560029 Bangalore, India

## Abstract

Pro-inflammatory and amyloidogenic S100A9 protein is an important contributor to Alzheimer’s disease (AD) pathology. Traumatic brain injury (TBI) is viewed as a precursor state for AD. Here we have shown that S100A9-driven amyloid-neuroinflammatory cascade was initiated in TBI and may serve as a mechanistic link between TBI and AD. By analyzing the TBI and AD human brain tissues, we demonstrated that in post-TBI tissues S100A9, produced by neurons and microglia, becomes drastically abundant compared to Aβ and contributes to both precursor-plaque formation and intracellular amyloid oligomerization. Conditions implicated in TBI, such as elevated S100A9 concentration, acidification and fever, provide strong positive feedback for S100A9 nucleation-dependent amyloid formation and delay in its proteinase clearance. Consequently, both intracellular and extracellular S100A9 oligomerization correlated with TBI secondary neuronal loss. Common morphology of TBI and AD plaques indicated their similar initiation around multiple aggregation centers. Importantly, in AD and TBI we found S100A9 plaques without Aβ. S100A9 and Aβ plaque pathology was significantly advanced in AD cases with TBI history at earlier age, signifying TBI as a risk factor. These new findings highlight the detrimental consequences of prolonged post-TBI neuroinflammation, which can sustain S100A9-driven amyloid-neurodegenerative cascade as a specific mechanism leading to AD development.

## Introduction

Over the past decade traumatic brain injury (TBI) has become the focus of increasing attention due to frequent incidences in modern society, including sport and military injuries. Despite extensive efforts to develop short and long-term neuroprotective strategies, these are not yet satisfactory and a better understanding of underlying pathologies is required to define the specific therapeutic targets. Following primary mechanical assault, TBI leads to delayed secondary responses at the molecular and cellular levels, which occur on a longer time scale and account for post-TBI neurological deficits^1^. There is growing epidemiological and clinical evidence that TBI incidences, including relatively mild injuries and even repetitive ball headings^2^, are strong risk factors for chronic traumatic encephalopathies and Alzheimer’s disease (AD)^3–5^.

Massive accumulations of amyloid-β peptide (Aβ) toxic oligomers and plaques^6^ are among the major AD pathological hallmarks and targets for therapeutic interventions. Inflammation also plays an important role in AD, which is supported by a sharp induction of inflammatory mediators in AD-affected brain^7^. Importantly, non-steroidal anti-inflammatory drugs may markedly reduce age-related prevalence of AD^8,9^ and slow amyloid deposition by mechanisms that still remain elusive^8^. Recently, we have found that pro-inflammatory mediator S100A9 can serve as a critical link between the amyloid cascade and neuroinflammatory events in AD^10^. Specifically, being highly amyloidogenic itself S100A9 can trigger and aggravate Aβ amyloid self-assembly and significantly contribute to amyloid cytotoxicity^10,11^. Both Aβ pathology^12,13^ and neuroinflammation^14,15^ are the key culprits in TBI secondary events, indicating that once these processes are initiated in TBI they can be further exacerbated in AD. Here we explore how this progression may occur with the focus on pro-inflammatory S100A9 and its role in the amyloid-neuroinflammatory cascade.

S100A9 is a multifunctional calcium-binding protein with diverse roles in the inflammatory signaling pathways. S100A9 belongs to the S100 protein family, which participates in a wide range of biological processes such as proliferation, migration and/or invasion, inflammation and differentiation^16–22^. S100 proteins, including S100A9, lack a signal peptide for secretion via the conventional Golgi-mediated pathway, and as whether extracellular S100 proteins are actively secreted from living cells via alternative secretion pathways or passively released is still debated^17,18,21^.

The increasing evidence indicates that S100A9, as well as other members of the S100 family, are pro-inflammatory molecules^22–25^. S100A9 was classified as damage associated molecular pattern (DAMP) molecule or alarmin broadly involved in infection, cellular stress, tissue damage and cancers^26–28^. Concerning intracellular functions of S100A9, there is evidence that S100A9 together with S100A8 interact in a calcium-dependent manner with cytoskeletal components^29^. Extracellular S100A9 is able to mediate cellular responses via receptors for advanced glycation endproducts (RAGE) or Toll-like 4 (TLR4) receptors, inducing expression of pro-inflammatory cytokines^26^. It has been shown that S100A9 and its fibrils regulate the NLRP3 inflammasome by acting as priming agents^30,31^.

A widespread expression of S100A9 was reported in many ailments associated with inflammatory processes, such as AD^10,32^, Parkinson’s disease^33^, malaria^34^, cerebral ischemia^35^, TBI^36^, obesity^37^ and cardiovascular disease^38^, implying that S100A9 may be a universal biomarker of inflammation. The abundance of S100A9 mRNA was also identified as a strong feature of aging in various mammalian tissues, including the central nervous system, and a novel mechanism of the age-associated inflammation sustained by S100A9 was suggested^39^. The distinctive feature of S100A9 compared to other pro-inflammatory mediators is its ability to self-assemble into amyloids following two-step nucleation-autocatalytic growth mechanism^40^, which may lead to the loss of its signaling functions and acquired amyloid cytotoxicity, exceeding that of Aβ^10^. Therefore, the rising S100A9 level during inflammation may lead to its amyloid formation and deposition as we have shown in AD^10^, aging prostate^41^ and also in cell model for protein amyloid aggregation^42^. Moreover, the CSF levels of S100A9 and Aβ match each other in AD, vascular dementia and mild cognitive impairment^43^, emphasizing the involvement of S100A9 together with Aβ in the amyloid-neuroinflammatory cascade in all these ailments. Interestingly, S100A9 knockdown attenuated memory impairment and reduced amyloid plaque burden in an AD mouse model^44^. However, intranasal administration of S100A9 oligomers and fibrils to wild-type mice induced wide-spread cellular stress responses and amyloid oligomerization in the brain tissues, as well as boosted hippocampal glutamate modifying monoaminergic neurochemistry, which all together led Alzheimer’s-like memory impairment in behavioural tests^45–47^. Similarly, in transgenic APP mouse model the amyloid aggregation of another protein from the S100 family – S100A8 has been shown to precede Aβ amyloid plaque formation and the positive feedback was found for both S100A8 and Aβ production^48^. Furthermore, S100A6 protein was also found to self-assemble into amyloid oligomers and fibrils, which can promote amyloid aggregation of superoxide dismutase-1 involved in amyotropic lateral sclerosis pathology^49^.

Here we have elucidated the role of S100A9 in the amyloid-neuroinflammatory cascades in TBI and compared with AD, viewing this as a common mechanism linking both ailments. We compared from this perspective the human brain tissues with TBI, AD, stable mild cognitive impairment (SMCI) and AD with TBI history. These studies were complemented by the primary neuronal cell culture experiments to demonstrate S100A9 induction at the cellular level under stress conditions. Moreover, at the molecular level the effects of acidification and elevated temperature on S100A9 amyloid formation and proteinase clearance were also examined *in vitro*. Consequently, those effects were linked with the resulting cytotoxicity of S100A9 amyloids. Thus, we have provided the comprehensive and systematic analysis of S100A9 role in TBI as a precursor state of AD at the molecular, cellular and tissue levels.

## Results

### Rapid formation of S100A9 and Aβ precursor-plaques in TBI

**Figure 1:**
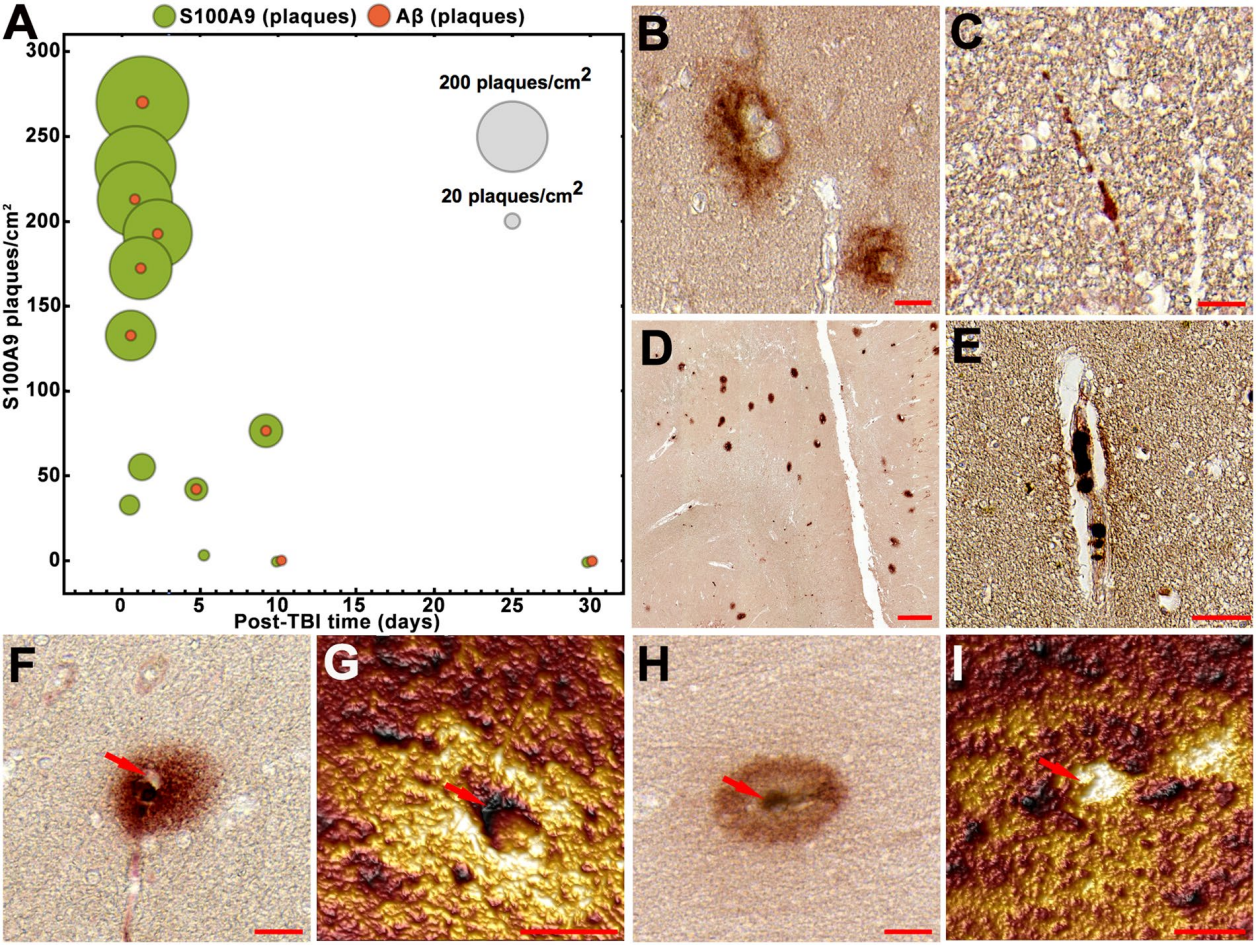
S100A9 and Aβ in TBI tissues. (**A**) Bubble chart showing counts of the precursor-plaques composed of Aβ (red) and S100A9 (light green), respectively, in the whole human TBI hippocampi and surrounding tissues in individual patients versus increasing post-TBI time (n = 13 TBI patients). Each bubble corresponds to the total counts in each individual patient tissue; the counts of S100A9 precursor-plaques per cm^2^ of tissue are plotted along *y*-axis and the scale bubbles are presented in grey. Representative images of Aβ immunopositive (**B**) precursor-plaques and (**C**) neuronal axons. Representative images of S100A9 immunopositive (**D**) precursor-plaques, (**E**) cells in blood vessel and (**F**,**H**) zoomed individual precursor-plaques. (**G**,**I**) AFM topographic images of S100A9 precursor-plaques shown in (**F**,**H**), respectively. Red arrows in (**F**–**I**) show the same position in optical and AFM images, respectively. Scale bars are 20 µm in (**B**,**F** and **H**), 50 µm in (**C**,**E**), 200 µm in (**D**) and 10 µm in (**G** and **I**). AFM z-heights correspond to a color gradient from 0 µm (dark brown) to 1.7 µm (yellow light).

The TBI brain tissues of 13 patients from 1 y.o. to 65 y.o. and with survival time from 4 h to 30 days (Supplementary Table S1) were subjected to immunohistochemical analysis using Aβ and S100A9 specific antibodies to examine the involvement of these antigens in TBI pathology. The Aβ immunopositive extracellular deposits were found in low numbers in 70% patients (Fig. 1A,B). These deposits were not reactive with amyloid oligomer (A11) and fibril specific (OC) antibodies (Supplementary Fig. S1), indicating the lack of amyloid structure in them, and, therefore, they were coined as precursor-plaques. Intracellular Aβ depositions were found in some axons (Fig. 1C) and they were also not reactive with A11 and OC antibodies (Supplementary Fig. S1).

By contrast, the immunostaining with S100A9 specific antibodies revealed that S100A9 precursor-plaques were highly abundant in all early TBI cases, including even the individuals who had no Aβ deposits at all, and their amounts were also by ca. 100 times higher than those of Aβ (Fig. 1A,D). The numbers of S100A9 plaques decreased to the level of Aβ deposits with increasing post-TBI time to ca. 4 days (Fig. 1A) and, likewise Aβ deposits, they were not reactive with A11 and OC antibodies (Supplementary Fig. S1). The S100A9 accumulation in macrophages and neutrophils^17,21,22^ was also found by S100A9 immunostaining in numerous blood vessels (Fig. 1E), which again did not show reactivity with A11 and OC antibodies (Supplementary Fig. S1). Generally, S100A9 precursor-plaques were not recognized by Aβ antibodies and *vice versa* as shown by the sequential immunohistochemistry experiments with a reverse order of S100A9 and Aβ antibodies, respectively (Fig. 1B, Supplementary Fig. S1).

In order to gain a higher resolution insight into the structure of S100A9 precursor-plaques, atomic force microscopy (AFM) topography imaging was conducted, matching the corresponding immunohistochemical images (Fig. 1F–I). Based on AFM topography we have distinguished two types of S100A9 precursor-plaques. The first type was characterized by a cavernous center (Fig. 1F,G), suggesting that S100A9 can be dispersed around damaged blood vessels. The second type showed an elevated AFM topographical profile in the center (Fig. 1H,I), indicating that precursor-plaque can possess a condensed core and does not originate from a blood vessel, but, for example, from damaged cells.

### Intracellular S100A9 and Aβ aggregation in TBI

**Figure 2:**
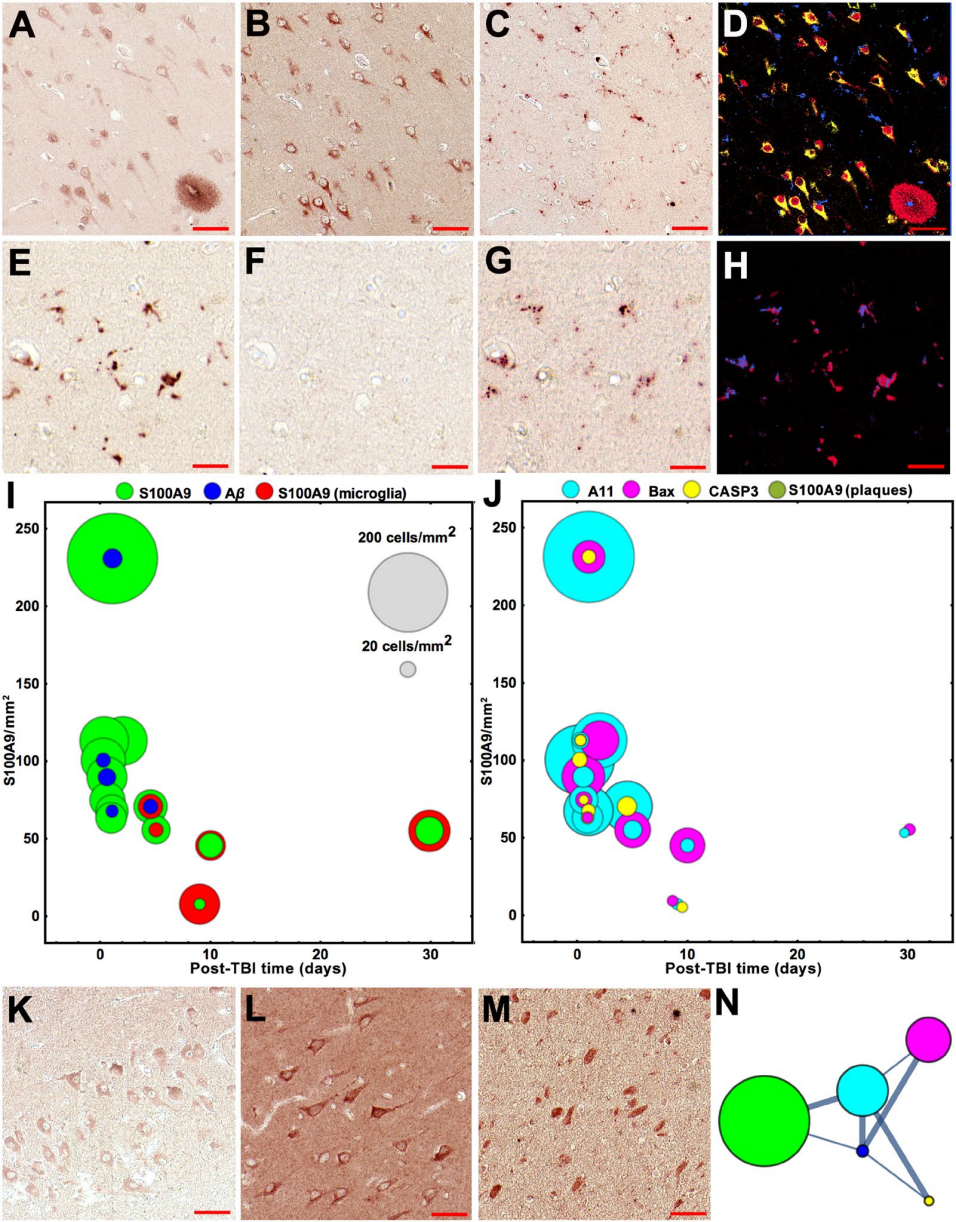
Analysis of intracellular S100A9/Aβ aggregation and apoptosis in TBI tissues. Representative images of sequential immunohistochemistry with (**A**) S100A9, (**B**) A11 and (**C**) CD68 antibodies (4 h post-TBI). (**D**) Superposition of the corresponding images in pseudo-colors, i.e. S100A9 staining is shown in red; CD68 – in blue and A11 – in yellow. Representative images of sequential immunohistochemistry of microglial cells with (**E**) S100A9, (**F**) A11 and (**G**) CD68 antibodies (5^th^ post-TBI day). (**H**) Superposition of the corresponding images in pseudo-colors, i.e. S100A9 staining is shown in red and CD68 – in blue. (**I**,**J**) Bubble charts showing the counts of immunopositive cells in the whole TBI hippocampi and surrounding tissues of individual patients upon increasing post-TBI time, n = 13 TBI patients. Each bubble corresponds to the total counts in individual patient tissue; the counts of S100A9 immunopositive neurons per mm^2^ are plotted along *y*-axis and the scale bubbles are presented in grey in (**I**). The counts for microglial cells reactive with S100A9 antibodies are shown in red, for neurons reactive with antibodies to S100A9 – in green, to Aβ – in blue, amyloid oligomers – in cyan, Bax – in magenta and activated caspase-3 – in yellow. Representativie imagies of immunostaining of neurons with (**K**) Aβ, (**L**) Bax and (**M**) activated caspase-3 antibodies. (**N**) Graph network, showing connections between the S100A9, Aβ, A11, Bax and activated caspase-3 positive neurons. Nodes correspond to the types of immunopositive cells and their color coding is indicated in (**I**,**J**). Node sizes are proportional to cell amounts. Nodes are connected by edges, whose widths reflect the correlation strength; thick lines show strong correlation and thin lines – moderate correlation. Values of Spearman’s rho correlations between immunopositive cell and plaque counts are presented in Supplementary Table S3. Scale bars are 50 µm in (**A**–**D** and **K**–**M**) and 20 µm in (**E**–**H**).

For the first time we have found that S100A9 was massively present and even formed amyloid oligomers in neurons already after 4 h following TBI: the same neurons were sequentially immunostained with both S100A9 and A11 antibodies (Fig. 2A,B,D). S100A9 was not detected in microglial cells at this post-TBI time, as evident from the sequential immunohistochemistry staining, where the S100A9 and CD68 immunostaining patterns did not overlap (Fig. 2A,C,D). Activated microglial cells expressed S100A9 only after 4 to 30 days of post-TBI, but no amyloid oligomers were formed within them, by contrast to neuronal cells, as demonstrated by the superposition of the S100A9 and CD68 immunostaining and the absence of A11 immunopositive pattern (Fig. 2E–H).

S100A9 and A11 immunopositive neurons were counted in the hippocampi and surrounding tissues of all TBI patients (Fig. 2I,J). With increasing post-TBI time their numbers significantly reduced and between 4 to 30 post-TBI days remained at the same level. During this post-TBI period the counts of S100A9 immunopositive microglial and neuronal cells were also similar (Fig. 2I). By contrast, Aβ immunopositive neurons were not detected in 8 out of 13 TBI cases and their numbers were by ca. 8-fold lower compared to S100A9 immunopositive neurons (Fig. 2I). Sequential immunohistochemistry revealed that some Aβ immunopositive neurons were also co-stained with S100A9 and A11 antibodies (Fig. 2K, Supplementary Fig. S2), indicating that Aβ and S100A9 can be co-localized and even co-aggregate together in neuronal cells under TBI. In the brain tissues of non-TBI controls neither S100A9 nor Aβ immunopositive staining was found (Supplementary Fig. S2).

### Neuronal apoptosis in TBI

Both excessive inflammatory cytokine production and amyloid oligomer formation can induce neuronal cell damage and death^50,51^. Here we examined the activation of apoptotic pathways by using immunohistochemistry with antibodies towards the apoptotic cascade markers – Bax and activated caspase-3^52,53^. Sequential immunohistochemistry revealed that some S100A9 and A11 immunopositive neurons were also stained with Bax or caspase-3 antibodies (Fig. 2L,M, Supplementary Fig. S2). Bax and activated caspase-3 immunopositive neurons were also counted in the hippocampi and surrounding tissues of all TBI patients (Fig. 2J). To identify the association between the intra-neuronal amyloid, neuroinflammatory and apoptotic events, Spearman’s rho correlations were calculated and the graph network was built up, demonstrating strong and moderate associations between them (Fig. 2N, Supplementary Table S3). The strong correlation (Spearman’s rho correlation coefficient equals 0.73) was found between intra-neuronal S100A9 presence and amyloid oligomerization, manifested in co-immunostaining with A11 antibodies. The correlation between intra-neuronal Aβ presence and amyloid oligomerization was also strong (0.62), despite the significantly lower number of Aβ immunopositive neurons. The moderate and strong associations were observed between the A11 and Bax (0.53) and between the A11 and activated caspase-3 (0.60) immunopositive neurons, respectively, demonstrating that the amyloid oligomerization may indeed prompt the activation of neuronal apoptotic pathways.

Interestingly, some S100A9 immunopositive neurons were found in the proximity to S100A9 precursor-plaques and the correlation between these events was also strong – with 0.65 Spearman’s rho correlation coefficient (Supplementary Table S3). This suggests that some of the S100A9 immunopositive cells may indeed contribute to the S100A9 precursor-plaque formation.

Importantly, the amounts of both S100A9 and Aβ precursor-plaques as well as the amyloid, neuroinflammatory and apoptotic responses in neurons and microglia, as reflected in the corresponding immunostaining patterns on a patient to patient basis, did not correlate with patient’s age (Supplementary Fig. S3), indicating that they are directly linked to TBI, rather than being age-dependent.

**Figure 3:**
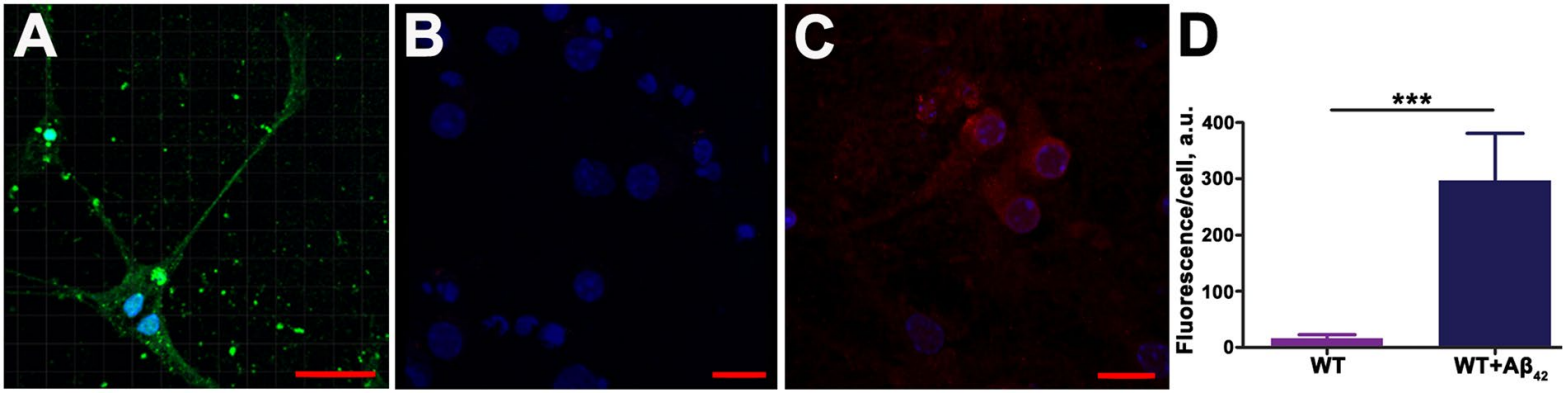
S100A9 expression in neuronal cell culture. (**A**) Fluorescence immunocytochemistry images by confocal microscopy of wild-type (WT) mouse primary neurons treated with Aβ_42_ oligomers. DAPI nuclei staining shown in blue and Aβ_42_ specific fluorescence – in green. Neurons before (**B**) and after Aβ_42_ oligomer treatment (**C**), respectively, with S100A9 content manifested in red fluorescence. (**D**) Quantification of S100A9 specific immunofluorescence signal per cell by using an Imaris software in untreated neurons (purple bar) and Aβ_42_ oligomer treated neurons (dark blue bar); the procedure is shown in Supplementary Fig. S7. Bars present means ± standard deviations in arbitrary units out of n = 7 and n = 6 measurements of untreated and treated cells, respectively. ***p < 0.0001 by Student’s *t* test. Scale bars are 30 µm (**A**), 15 µm (**B**) and 20 µm (**C**).

The fact that S100A9 can be quickly induced in neurons was confirmed also in wild-type mouse primary neuronal cell culture upon the limited exposure of neurons to extracellular stress factor, specifically to Aβ_42_ oligomers (Fig. 3A). If before treatment the immunofluorescence of S100A9 antibodies in neuronal cells was close to noise level (Fig. 3B), it significantly increased within 24 h following addition of Aβ_42_ oligomers to cell culture (Fig. 3C). The relative quantification of S100A9 level in neurons was performed by an Imaris software in confocal images and demonstrated significant raise of S100A9-specific immunofluorescence intensity in neurons from the noise level to ca. 300 fold stronger signal per cell after the cellular stress induced by Aβ_42_ oligomers (Fig. 3D). This is congruous with trauma-associated S100A9 neuronal induction in the post-TBI human brain tissues.

### S100A9 specific amyloid plaques in SMCI and AD

**Figure 4:**
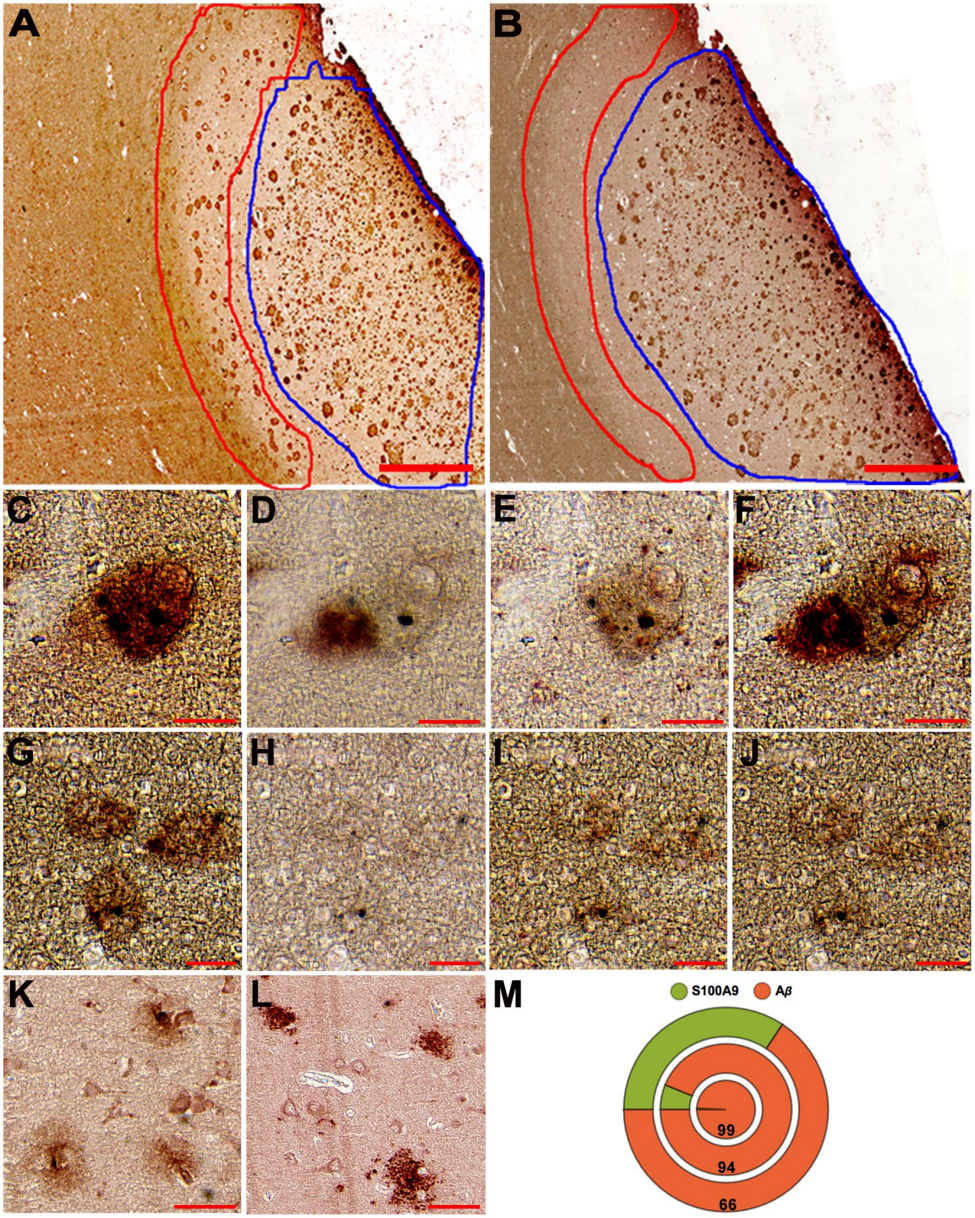
S100A9 and Aβ plaques in human AD and SMCI tissues. n = 6 AD cases and n = 1 SMCI case. (**A**,**B**) Representative immunostaining with S100A9 and Aβ antibodies, respectively, of the grey and white matter areas in the frontal lobe of AD patient. The regions with S100A9 plaques at their interface and mature Aβ-S100A9 senile plaques within the grey matter are encircled by red and blue lines, respectively. Representative sequential immunohistochemistry of conjugated Aβ-S100A9 amyloid plaque with (**C**) S100A9, (**D**) Aβ, (**E**) A11 and (**F**) OC antibodies, respectively. Representative S100A9 plaques stained with sequence of (**G**) S100A9, (**H**) Aβ, (**I**) A11 and (**J**) OC antibodies, respectively. Representative sequential immunostaining of amyloid plaques in SMCI frontal lobe with (**K**) S100A9 and (**L**) Aβ antibodies, respectively. (**M**) Pie chart showing the amount of S100A9 (green) and Aβ (red) immunopositive plaques in SMCI (n = 1) and AD (n = 2) patients. Each circle corresponds to individual subject; outer circle shows the distribution of plaques in SMCI case and two inner circles – in two AD cases; numbers in the red circles indicate the percentage of Aβ plaques in the corresponding case. Scale bars are 500 µm in (**A**,**B**), 25 µm in (**C**–**J**) and 50 µm in (**K**,**L**).

In all 6 AD cases examined here we have found that most of senile plaques contained not only commonly expected Aβ, but also S100A9 as we have shown previously^10^. Indeed, S100A9 and Aβ immunostaining patterns were overlapped as shown over the large brain tissue area enriched with plaques and encircled by blue line (Fig. 4A,B) and in the magnified views of particular amyloid plaques (Supplementary Fig. S4). The Aβ-S100A9 plaques were also reactive with OC antibodies, demonstrating their amyloid nature (Supplementary Fig. S4). However, we have observed that within some plaques the S100A9 and Aβ immunostaining patterns were not overlapping as shown previously^10^, but located adjacently to each other (Fig. 4C–F), suggesting that both polypeptides may play an autonomous role in the plaque formation.

Moreover, in 50% AD cases we have found specific type of amyloid plaques, which were reactive only with S100A9, but not Aβ antibodies (Fig. 4G,H, Supplementary Fig. S4). The S100A9 plaques were usually stained with both A11 and OC amyloid-specific antibodies (Fig. 4G,I,J) or sometimes only with A11 antibodies (Supplementary Fig. S4), thus displaying the amyloid character with different degree of amyloid maturation from oligomers to fibrils. Generally, the S100A9 plaques were randomly distributed among the Aβ-S100A9 senile plaques, but in 1 AD patient they were located with high density in distinct area at the border of the white and grey matters (Fig. 4A,B). In addition, in 1 SMCI patient, who has not yet develop AD and who had received multiple TBIs during his Ukrainian military service, we also observed distinct S100A9 and Aβ plaques, which were not cross-reactive with both S100A9 and Aβ antibodies, as well as plaques containing both antigens (Fig. 4K,L). In this SMCI patient the S100A9 immunopositive plaques not reactive with Aβ antibodies constituted 34% of all plaques (Fig. 4M), while in AD patients these numbers were 1 and 6%, the latter corresponded to AD patient with TBI history. This indicates that with progression of neurodegeneration the involvement of Aβ in plaque formation increases, but previous TBI history can be manifested in significant depositions of S100A9 alone already at the stage of SMCI. Importantly, the SMCI (43 y.o.) and AD (57 y.o) cases, both characterized by TBI history, displayed the advanced plaque pathology at relatively early age compared to an average age for other AD patients without known TBI history (82.6 y.o) (Supplementary Table S1).

**Figure 5:**
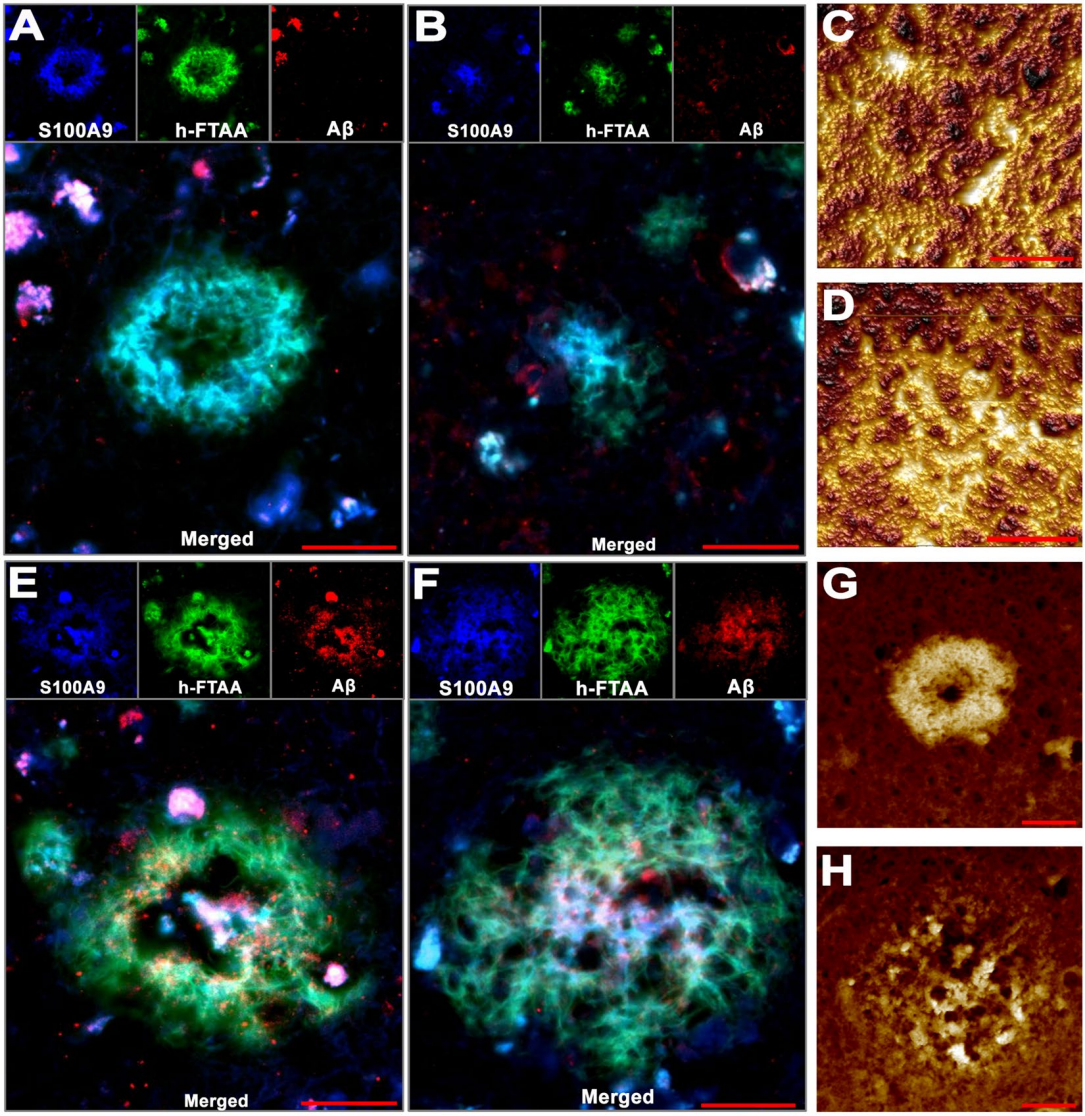
S100A9 and Aβ-S100A9 senile plaque structure in AD. n = 3 AD patient brain tissues were analyzed. (**A**,**B**) Immuno- and h-FTAA fluorescence imaging of two representative S100A9 amyloid plaques. Single channel images are shown at the top and merged images – at the bottom. S100A9 antibody staining is shown in blue, h-FTAA – in green and Aβ – in red. (**C**,**D**) AFM topography images of two representative S100A9 amyloid plaques. (**E**,**F**) Immuno- and h-FTAA fluorescence imaging of two representative Aβ-S100A9 amyloid plaques. (**G**,**H**) AFM topography images of two representative Aβ-S100A9 amyloid plaques. Scale bars are 20 µm in (**A**,**B**,**E**–**H**) and 10 µm in (**C**,**D**). AFM z-heights correspond to a color gradient from 0 µm, shown in dark brown, to (**C**) 1.8 µm, (**D**,**H**) 3.3 µm and (**G**) 3.8 µm shown in yellow light, respectively.

To gain insight into the structures of only S100A9 (Fig. 5A–D) and joined Aβ-S100A9 (Fig. 5E–H) plaques, the combined immunofluorescence with S100A9 and Aβ antibodies, h-FTAA fluorescence and AFM topographic imaging were performed. The fluorescence images of both types of plaques showed that they were reactive with amyloid specific h-FTAA dye^54^, indicating their amyloid nature, and characterized by a patchy distribution of amyloid materials as evident from the corresponding patchy staining patterns (Fig. 5A,B,E,F). Interestingly, in both types of plaques the amyloid material can be deposited either at their circumference, forming donut-like structures (Fig. 5A,C,E,G) or spread from their centers (Fig. 5B,D,F,H), as shown by both fluorescence (individual and superimposed colored patterns) and AFM techniques (deposited material is shown in yellow light color). These structural similarities suggest the common mechanisms of formation for both types of plaques composed solely of S100A9 and both S100A9-Aβ.

### Modelling S100A9 amyloid formation, seeding and disaggregation *in vitro*

The amyloid accumulation in the brain tissues depends on both amyloid growth and clearance, including proteolytic cleavage. Alzheimer’s amyloid plaques are very protease resistant aggregates^55^. *In vitro* studies demonstrated that the insulin amyloid protease resistance is modulated by environmental conditions and amyloid aging^56^. Here we have examined the effect of acidification and increasing temperature, which are both relevant to traumatic brain injury conditions^57,58^, on S100A9 amyloid formation and proteinase disaggregation.

**Figure 6:**
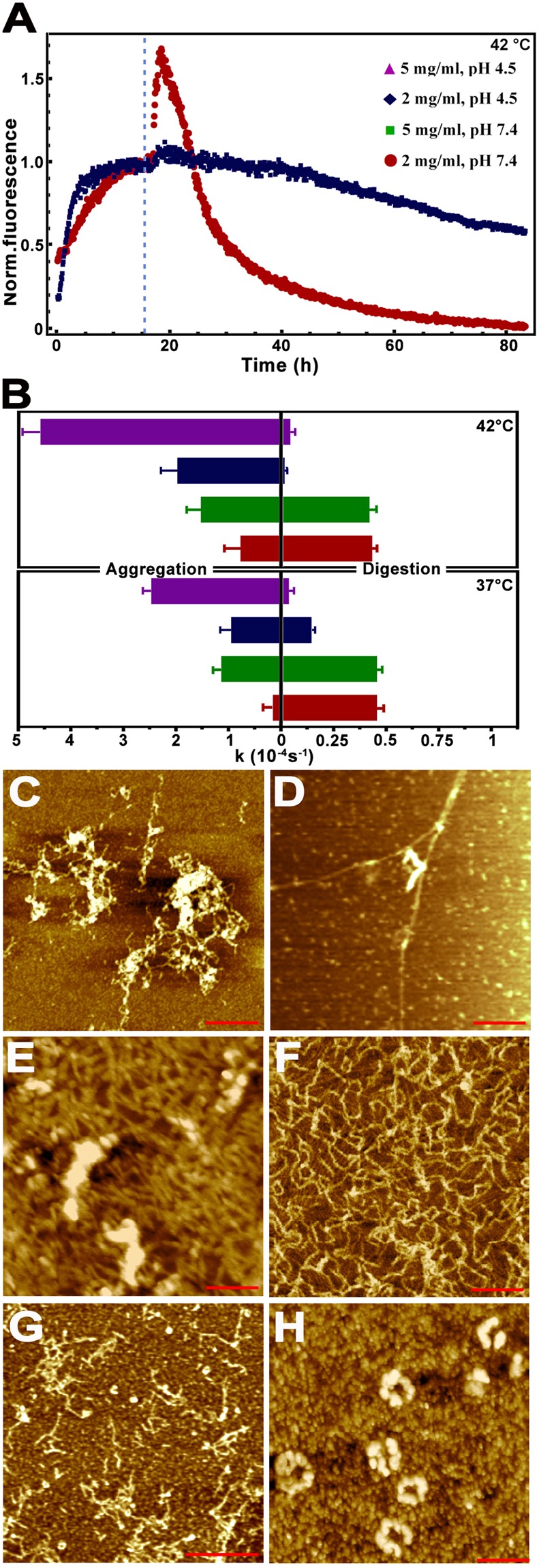
*In vitro* S100A9 amyloid formation and proteinase K digestion. (**A**) Kinetics of S100A9 amyloid formation and proteinase K digestion monitored by h-FTAA fluorescence at 42 °C. Time of protease K addition is indicated by dashed line. (**B**) Rate constants of S100A9 amyloid formation (left) and proteinase K digestion (right), shown by bars and corresponding to 42 °C (top) and 37 °C (bottom), represent mean ± standard deviation (n = 10 repeats for each kinetic measurement). Kinetic data observed at 5 mg/ml, pH 4.5 is shown in purple, at 2 mg/ml, pH 4.5 – in dark blue, 5 mg/ml, pH 7.4 – in green and 2 mg/ml, pH 7.4 – in red, as indicated in figure legend (**A**). Representative AFM height images of (**C**) S100A9 amyloid clumps formed in 10 mM PBS, pH 7.4, 37 °C, during 12 h under shaking with glass beads; (**D**) the same sample after 6 h proteinase K digestion. Representative AFM height images of (**E**) S100A9 amyloid clumps and fibrils formed in 20 mM sodium acetate, pH 4.5, 42 °C, during 8 h under shaking with glass beads; (**F**) the same sample subjected to 3 h, (**G**) 6 h and (**H**) 12 h proteinase K digestion. Scale bars are 250 nm in (**C**–**F**) and 600 nm in (**G**,**H**), respectively.

The S100A9 amyloid formation and proteinase K disaggregation kinetics *in vitro* were monitored by h-FTAA amyloid fluorescence assay in conjunction with AFM (Fig. 6, Supplementary Fig. S5). All S100A9 amyloid kinetics at different protein concentrations (2 and 5 mg/ml), pH (7.4 and 4.5) and temperatures (37 and 42 °C) were well fitted by the nucleation-dependent polymerization model^59^ (Fig. 6A, Supplementary Fig. S5). Following the nucleation, the amyloid assembly resulted in massive amyloid fibril formation, which were observed at time corresponding to the kinetic plateau levels, and were observed by AFM (Fig. 6C,E). The amyloid assembly at pH 4.5 and 42 °C was characterized by the highest rate constants compared to the rate constant observed at pH 7.4 and 37 °C, as well as compared to pH 7.4, 42 °C and pH 4.5, 37 °C conditions, signifying the role of acidification and raised temperature in the S100A9 amyloid formation (Fig. 6B).

After 16 h incubation the S100A9 amyloid samples described above were subjected to proteinase K cleavage (Fig. 6A). This followed by an initial rise of h-FTAA fluorescence above the kinetic plateau level, which may be due to increased number of nuclei produced by proteinase K cleavage^56^. This spike was very pronounced but short lived at pH 7.4, 37 °C, when S100A9 amyloids became rapidly digested as evident both from the drop of h-FTAA fluorescence and AFM observations of their progressing disappearance after 6 and 72 h, respectively (Fig. 6D, Supplementary Fig. S5). By contrast, the proteinase K digestion of S100A9 amyloids at pH 4.5, 42 °C was much slower as evident from the lower rates of amyloid digestion kinetics (Fig. 6B), higher level of remaining h-FTAA fluorescence (Fig. 6A) and the presence of amyloids after proteinase K treatment as observed in AFM images (Fig. 6F–H, Supplementary Fig. S5). Massive S100A9 amyloid fibrils became thinner and shorter during 3 to 6 h proteinase K digestion (Fig. 6F,G), however a large quantity of ring-shaped amyloid assemblies and oligomers were formed and still present after 12 and even 72 h (Fig. 6H, Supplementary Fig. S5). This suggests that the perturbation of tissue homeostasis in the post-TBI brain manifested in its acidification and elevated temperature^55,57^ as well as in the significant increase of S100A9 concentration may be of a major importance in both promoting undesirable S100A9 amyloid self-assemblies, slowing their clearance and even inducing their structural rearrangement into more toxic amyloid species (Fig. 6H), which all together can be more harmful.

Since S100A9 but not Aβ was abundantly present in the precursor plaques and intracellularly in TBI tissues, the effect of S100A9 pre-formed fibrils on Aβ_42_ aggregation was examined *in vitro* (Supplementary Fig. S6). We have observed significant seeding effect on Aβ_42_ amyloid formation induced in a concentration-dependent manner by pre-formed amyloid fibrils of S100A9. These reflected in shortening of lag-phase of the Aβ_42_ amyloid kinetic curve from >2 h in the absence of S100A9 fibrils to ca. 1.4 h in the presence of 30 μM of S100A9 fibrillar sample, while the plateau was reached also faster, i.e. after 4.5 h in the absence and 2.4 h in the presence of 30 μM S100A9 fibrillar sample, respectively.

### S100A9 amyloid cytotoxicity

**Figure 7:**
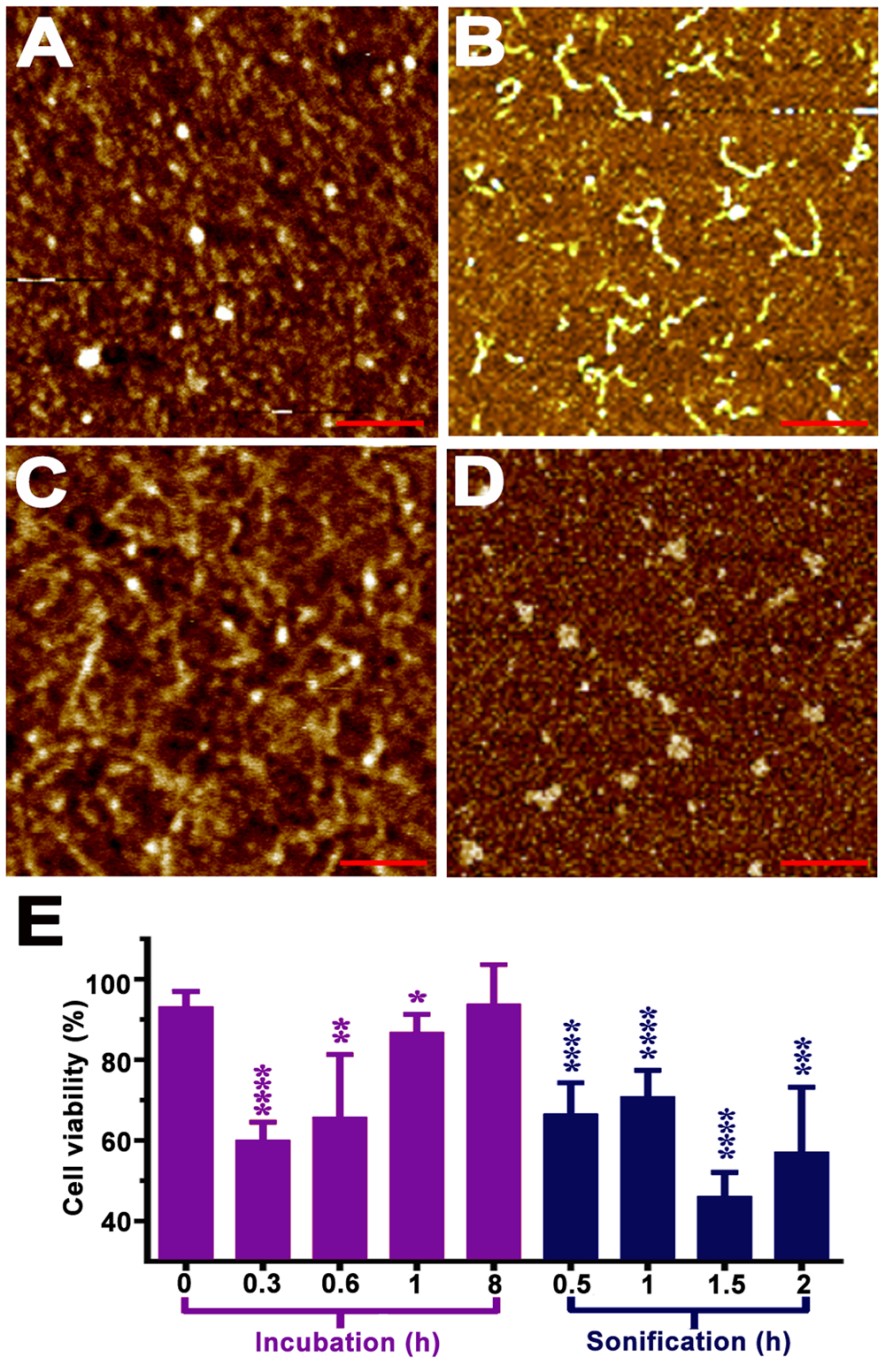
S100A9 amyloid cytotoxicity. Representative AFM height images of (**A**) S100A9 amyloid oligomers formed after 0.3 h and (**B**) protofilaments formed after 1 h incubation in 20 mM sodium acetate, pH 4.5, 42 °C under shaking with glass beads; (**C**) S100A9 amyloids, incubated for 8 h at the above conditions, were subjected to 0.5 h and (**D**) 1.5 h sonication. (**E**). Viability of SH-SY5Y cells measured by WST-1 assay after 24 h co-incubation with 20 μM S100A9 amyloids shown in (**A**–**D**). The cell viability depending on the added amyloid pre-incubation time is shown in magenta and the cell viability upon pre-formed amyloid sonication time – in blue. The viability of cells with added buffer is taken as 100%. Cell viability is shown by mean ± standard deviation, n = 7 repeats of each measurement. *p = 0.023, **p = 0.0023, ***p = 0.0004, ****p < 0.0001, calculated by Student’s *t* test.

The cytotoxic properties of S100A9 amyloid aggregates produced at pH 4.5, 42 °C were examined by adding them to SH-SY5Y neuroblastoma cells (Fig. 7). The amyloid species were collected after 0.3, 0.6, 1 and 8 h incubation periods to sample the amyloid assemblies progressing from oligomers to fibrils in accord with the kinetics of S100A9 amyloid formation presented in Fig. 6A and Supplementary S5, i.e. the oligomers were populated in the beginning of the amyloid formation process and fibrils were developed when the plateau level of the kinetic curve was reached. The morphology of S100A9 amyloid structures produced during the course of incubation at the above conditions were also examined by AFM prior subjecting them to cytotoxicity experiments and their images are presented in Fig. 7A–C. Freshly dissolved native S100A9 (zero time point) was not cytotoxic within the error of measurement (Fig. 7E). Short protofilaments and oligomers populated after 0.3 h amyloid incubation (Fig. 7A) were the most toxic, reducing the cell viability by 40%, as the amyloid oligomers are viewed to be the most cytotoxic species^60^. S100A9 amyloid cytotoxicity subsided, however, when filamentous structures were developed upon further incubation (Fig. 7B), and mature fibrils formed after 8 h incubation were not toxic, when added to cells.

To model the S100A9 amyloid fragmentation process occurring during proteinase K digestion, its amyloids were subjected to sonication (Fig. 7C,D). The SH-SY5Y cell viability in the presence of sonicated S100A9 protofilaments and oligomers (Fig. 7C), including ring-shaped assemblies (Fig. 7D), which are viewed to be the most toxic^10^, reduced by ca. 30 and 50%, respectively. This suggests that amyloid proteinase digestion indeed can potentially produce secondary highly toxic S100A9 amyloid species.

## Discussion

The main goal of this research is to demonstrate that S100A9, in addition to commonly perceived Aβ peptide, may be a leading causative component of the amyloid-neuroinflammatory cascade, triggered in TBI and presenting the risk for AD and other neurodegeneration disease development. The amyloid cascade is central for understanding the molecular and cellular pathology of AD^6^, however there is a lack of data to demonstrate how this cascade is initiated. If to examine the advanced AD brain tissues, it is not possible to conclude beyond doubt as whether Aβ, but not some other factors, were at the origin of disease. This is why we have examined TBI, which is considered as a precursor state or risk factor for AD^12,51,61^. Previously TBI was linked to AD via the amyloid cascade and aggregation of Aβ peptide. However in the present research we have not found massive Aβ presence either in the precursor plaques or within the neuronal cells after TBI. Conversely, we have found an abundance of S100A9 both extracellularly in the precursor plaques and within neurons and microglial cells. Acute and chronic inflammation associated with TBI may lead to rising S100A9 level, especially as the latter can be sustained over years following TBI^1,3,62^. Moreover we have shown for the first time that in AD there are areas which contain only S100A9 plaques compared to those, which encompass both Aβ and S100A9. Therefore we demonstrated that S100A9 can be a common denominator in inflammation-associated conditions in TBI and AD as a driving component of the amyloid-neuroinflammatory cascade.

Previously it was suggested that the AD amyloid pathology can be initiated within hours after TBI, that manifested in appearance of Aβ plaques in ca. 30% acute cases^12,61^. We have demonstrated that S100A9 is dominant compared to Aβ both intra and extracellularly in post-TBI tissues, indicating that S100A9, but not Aβ, may play a leading role in amyloid aggregation. The dominance of S100A9 precursor-plaques was found in all TBI cases *versus* Aβ depositions in ca. 70% individuals (Fig. 1A). Moreover, on a patient to patient basis the amounts of S100A9 precursor-plaques were also overwhelmingly higher compared to those of Aβ, i.e. by 100-fold. We have not found any correlation between the accumulation of S100A9 and Aβ precursor-plaques and age, highlighting that this phenomenon is the consequence of TBI, but not ageing (Supplementary Fig. S3). In all individuals both S100A9 and Aβ precursor-plaques were relatively new lesions not reactive with amyloid-specific antibodies and dye h-FTAA, as they were developed on a time scale much shorter than required for AD senile plaque formation. This is consistent with previously reported lack of Congo red birefringence of TBI Aβ plaques, questioning their amyloid nature^63^, otherwise the amyloid character of Aβ deposits was not examined at all^13^. The staining of Aβ plaques with amyloid specific thioflavin S dye was observed only in post-TBI with long history (1 to 47 years)^12^. We have shown that the number of S100A9 precursor-plaques reduced to the level of Aβ deposits with increasing post-TBI time. Since pro-inflammatory S100A9 is highly amyloidogenic and its fibrils are able to seed the amyloid formation of Aβ_42_ as shown *in vitro* (Supplementary Fig. S6), if chronic inflammation would persist, sustaining its elevated level, its precursor-plaques may remain and seed further amyloid growth, representing a serious risk for AD development.

For the first time we have found specifically S100A9 amyloid plaques, which did not contain Aβ, in both SMCI with TBI history and half of AD cases, including the AD case with TBI history (Fig. 4). Remarkably, in SMCI the S100A9 plaques constituted as many as third of all deposits. Importantly, in SMCI and AD with TBI history the advanced plaque pathology was also developed at earlier age compared to an average age of the AD patients without TBI history. In the light of the amyloid hypothesis, these facts once again emphasise that high production and aggregation of S100A9 triggered by primary TBI insult, either in combination with Aβ or on its own, may provide a template for further senile plaque development leading to AD.

In AD we have shown the similarity of the plaques containing only S100A9 and mixed Aβ-S100A9 senile plaques with regards to their (*a*) amyloidogenic properties, such as staining with amyloid-specific h-FTAA dye, A11 and OC antibodies, and (*b*) morphological features shown by AFM (Fig. 5). Both types of AD plaques as well as precursor-plaques in TBI were characterized by patchy or diffused depositions of proteinaceous material as revealed by AFM, fluorescence microscopy and immunohistochemistry (Figs 1 and 5). They all displayed also similar topography, where the condensation of proteinaceous material occurred either at the circumference or spread from the center, indicating that all plaques (S100A9 and Aβ-S100A9) may originate via similar deposition mechanisms.

We have shown that the high abundance of S100A9 in TBI tissues was manifested in its intraneuronal and microglia presence (Fig. 2). The level of S100A9 immunopositive cells in TBI tissues was much higher than those of Aβ, i.e. (*a*) Aβ immunopositive neurons were found in only ca. 40% TBI cases and (*b*) their amounts were by 10-fold lower. These again highlight that the sustained high level of amyloidogenic protein in TBI is likely linked to S100A9, but not to Aβ.

By using sequential immunohistochemistry and graph analysis, we have shown that S100A9 intraneuronal oligomerization is an essential factor promoting the apoptotic pathway via Bax and caspase-3 activation (Fig. 2), which may play a major role in TBI secondary neuronal loss^64^. Since the number of neurons with Aβ peptide is much lower than those containing S100A9 (Fig. 2I), the potential mechanism of secondary neuronal loss in TBI is also more likely to be associated with S100A9 oligomerization and not Aβ.

The perturbations of brain tissue homeostasis manifested in acidification and fever were implicated in post-TBI, especially in the cases with unfavorable neurological outcome^57,58^. Here we demonstrated that S100A9 readily forms amyloids *in vitro* under variety of TBI relevant conditions, including increased protein concentration, acidic pH and elevated temperature, and these factors provide a strong positive feedback to amyloid self-assembly (Fig. 6). The S100A9 oligomers can be highly toxic if they are added to neuroblastoma cells (Fig. 7). Therefore S100A9 oligomerization, occurring both extra and intracellularly very rapidly after TBI, can be a target for potential therapeutic interventions aiming to prevent their harmful effects. Prospective anti-inflammatory medications can be easily implemented in post-TBI to achieve this goal^9^.

At the same time, acidification and raising temperature slowed proteinase K digestion of S100A9 amyloids, resulting in the prolonged life-span of short and cytotoxic amyloid species (Fig. 6), which can again cause the secondary neuronal loss. Altogether the *in vitro* experiments demonstrated how the environmental factors implicated in TBI paired with high expression of S100A9 in TBI tissues can produce unfavorable outcomes such as S100A9 amyloidogenicity and neuronal cytotoxicity.

**Figure 8:**
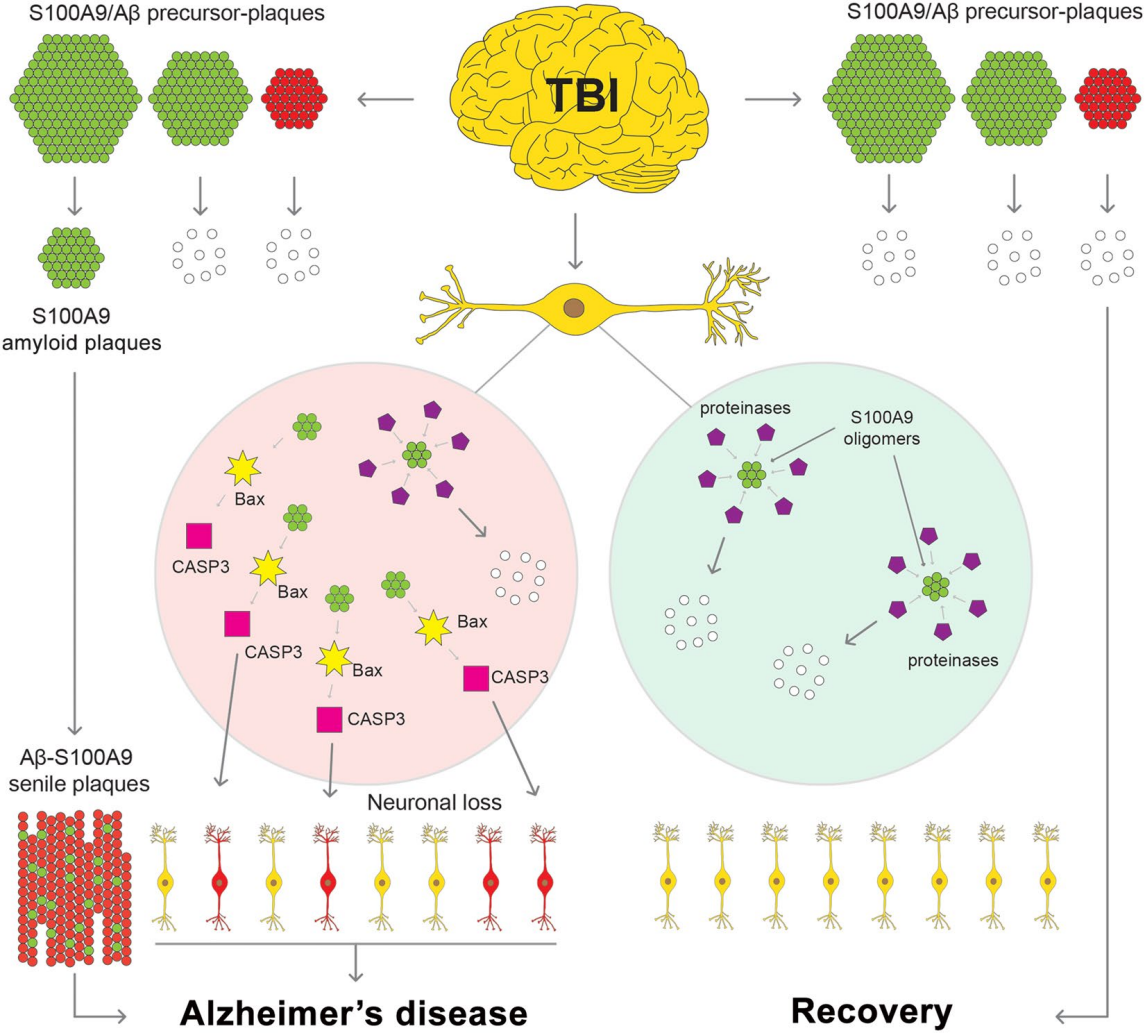
Schematic presentation of S100A9-driven amyloid-neuroinflammatory cascade in TBI. Most of S100A9 precursor-plaques (shown schematically in green) and Aβ precursor-plaques (shown in red) formed during TBI will undergo clearance (white), leading to recovery from TBI. However, some S100A9 and Aβ precursor-plaques will develop into AD senile plaques (green-red). Intracellular S100A9 oligomers (green), if they are not cleared by proteases (magenta) and other cellular clearance mechanisms, can trigger the apoptotic cascade involving Bax and activated caspase-3, leading to neurodegeneration and AD.

The summary of our findings is schematically outlined in Fig. 8. This highlights both intra and extracellular pathways of S100A9 and Aβ amyloid self-assembly, which are intertwined together leading to cell death and neurodegeneration. For the first time we have presented here a compelling evidence for the critical role played by pro-inflammatory S100A9 protein in the amyloid-neuroinflammatory cascade in TBI, which resembles similar processes in AD and may serve as a mechanistic link between TBI and AD. While the topic of inflammation in neurodegeneration is well discussed, here we present specific culprit – S100A9, and specific mechanism – S100A9 amyloid formation, which can drive TBI-induced inflammation to the amyloid cascade in Alzheimer’s disease. Therefore, S100A9 can be viewed as a prospective therapeutic target during various post-TBI stages and far prior to AD development to halt and reverse these damaging processes.

## Methods

### Regulatory Compliance

The Medical Ethics Committees of Umeå University Hospital, Sweden; Human Brain Tissue Repository for Neurobiological Studies, National Institute of Mental Health and Neurosciences, Bangalore, India; Institute of Neurology, Medical University of Vienna, Austria and Medical Institute, Sumy State University, Ukraine approved all procedures detailed in this study, that required the use of human brain tissues prior to initiating any experimental objectives. Additionally, all methods were performed in full compliance with the Medical Ethics Committees of Umeå University Hospital, Sweden; Human Brain Tissue Repository for Neurobiological Studies, National Institute of Mental Health and Neurosciences, Bangalore, India; Institute of Neurology, Medical University of Vienna, Austria and Medical Institute, Sumy State University, Ukraine. The surrogates of all patients gave informed consent for the use of patient’s post-mortem tissue samples for research studies.

### Human tissues

Brain tissues from 13 TBI, 6 AD and 1 SMCI as well as 3 non-demented controls were examined. TBI patients were from 1 to 65 y.o. and without any AD history. Older patients were excluded from this study due to age-dependent neurodegenerative changes. 1 AD and 1 SMCI patients had TBI history. All tissues were paraffin embedded and microtome-sectioned to 4 µm thick slices. The characteristics of patients, including their age, sex, diagnosis and post-TBI time, if relevant, are presented in Supplementary Table S1.

### Materials

S100A9 was expressed in *E coli*, purified as described previously and its concentrations were determined by using *ε*_280_ = 0.53 (mg/ml)^−1^ cm^−1^ ^65^. Aβ_42_ was purchased from Tocris Bioscience.

### Conditions of amyloid formation and proteinase K digestion

In order to produce amyloid structures S100A9 was incubated at 2 and 5 mg/ml concentrations in either 20 mM Na acetate, at pH 4.5 or 10 mM phosphate buffered saline (PBS), containing 140 mM NaCl, 2.7 mM KCl, 10 mM Na_2_HPO_4_ and 2 mM KH_2_PO_4_, at pH 7.4. All samples were incubated both at 37 or 42 °C. Proteinase K was added to all these amyloid samples after 16 h incubation at 1:400 molar ratio to S100A9 and this reaction was stopped after 70 h. Shaking at ca. 200 rpm with glass beads in a Tecan plate reader was used for all these samples. S100A9 amyloid samples produced at 5 mg/ml, in 20 mM Na acetate, pH 4.5, 42 °C during 12 h were also sonicated from 0.5 to 2 h in a Transsonic T310 and subjected to the cytotoxicity measurements. Aβ_42_ added to neuronal cell culture was dissolved in 10 mM NaOH and centrifuged at 15000 rpm for 30 min at 4 °C to remove aggregates. Supernatant was collected and 10 mM PBS, pH 7.4 was added to produce required final concentration determined by both Bradford assay and absorbance at 220 nm^66^. Aβ_42_ oligomers were produced at 100 µM concentration after 30 min incubation at room temperature.

### Immunohistochemistry

Single and sequential immunohistochemistry with the same tissue sections was performed as described previously^10^, by using reagents and antibodies summarized in Supplementary Table S2. The corresponding immunopositive plaques and cells were manually counted in the entire hippocampus region and surrounding areas.

### AFM

Brain tissues and *in vitro* produced amyloid samples were imaged by a Bruker Bioscope Catalyst microscope operated in the peak force mode at 1 kHz frequency with 6 N/m stiff cantilevers. TBI and AD tissue samples were deparaffinized, dehydrated and immunostained with corresponding antibodies. The tissue sections were kept on the glass slides during all above procedures and AFM scanning. *In vitro* produced amyloid samples were deposited on the surface of freshly cleaved mica (Ted Pella) for 15 min, washed 3 × 100 μl by deionized water and dried at room temperature.

### Immuno- and h-FTAA fluorescence of human brain tissues

Brain tissue sections from the TBI and AD subjects were deparaffinized, dehydrated, immunostained with antibodies towards S100A9 and Aβ and then subjected to 30 min incubation with 0.3 µM h-FTAA fluorescent dye^54^, which specifically binds to amyloids. Then the tissue sections were washed with PBS and mounted on glass slides. Fluorescence images were recorded by a DM6000 B fluorescence microscope (Leica Microsystems) equipped with a SpectraCube module (Applied Spectral Imaging) and bandpass filters 436/10 (LP475) – blue, 535/50 (LP590) – green and 640/30 (BP 700/75) – red. The reagents and antibodies are outlined in Supplementary Table S2.

### h-FTAA fluorescence *in vitro*

h-FTAA was used to monitor *in vitro* the kinetics of amyloid formation and its digestion by proteinase K. Fluorescence of 0.3 µM h-FTAA was recorded by a Tecan P-200 fluorescence plate reader using excitation at 485 nm and emission at 590 nm.

### Kinetics data fit of S100A9 amyloid aggregation and proteinase K digestion

S100A9 amyloid kinetics were described by nucleated polymerization model with some modification, accounting for the presence of pre-formed seeded aggregates^59^. Each data set was repeated 10 times and averaged. The kinetics were baseline-corrected and normalized, such that the relative mass concentration of amyloid aggregates was *m(0)* at time 0 and 1 at completion of the aggregation. The mass concentration of amyloid polymers in solution *M(t)* was described by eq. (1) from which the effective rate constant *λ* and nuclei size *n*_*c*_ were derived by using the best fit.

1 $$M(t)={m}_{tot}-m(0){[\mu {\rm{s}}{\rm{e}}{\rm{c}}{\rm{h}}(\nu +\lambda {\beta }^{-\frac{1}{2}}\mu t)]}^{\beta },$$

where *m*_*tot*_ is total mass concentration of non-aggregated protein; *m(0)* – initial mass concentration of amyloid seeds; *λ –* effective rate constant, which was defined as $\lambda =\sqrt{2{k}_{n}{k}_{+}m{(0)}^{{n}_{c}}}$, *k*_+_ – elongation rate and *k*_*n*_ – nucleation rate; *β* = 2/*n*_*c*_, $\mu =\sqrt{1+{\gamma }^{2}}$, $\nu =\arcsin (\gamma )$ and $\gamma =\frac{2{k}_{+}P(0)}{{\beta }^{\frac{1}{2}}\lambda }$ where *P(0)* – initial number of pre-aggregated seeds.

Amyloid proteinase K digestion processes were described by single exponential decays

2 $$m(t)=A{e}^{-kt},$$

where *m(t)* is mass concentration of digested amyloids, *A* – arbitrary amplitude and *k* – digestion rate.

### Cytotoxicity assay

Viability of SH-SY5Y neuroblastoma cells was measured by WST-1 assay after 24 h co-incubation with amyloids as described previously^10^. SH-SY5Y cells were cultured in Dulbecco’s modified Eagle’s medium supplemented with 10% (v/v) fetal bovine serum and antibiotics in a 5% CO_2_ humidified atmosphere at 37 °C. Cells were plated at a density of 104 cells/well in 96-well plates; after 24 h of incubation, the medium was changed before incubation with amyloid samples. Initially S100A9 amyloid samples were incubated at 400 μM in 20 μM sodium acetate buffer, pH 4.5, 42 °C with shaking during different time periods (0, 0.3, 0.6, 1, and 8 h) to produce oligomers and fibrils, respectively. Then S100A9 samples were diluted in the culture medium and added to SH-SY5Y cells at a final concentration of 20 μM. The cell samples were co-incubated with S100A9 specimens for 24 h prior the cell viability was assessed by WST-1 assay. 10 μl of WST-1 reagent (Roche, Germany) was added to 100 μl of cell culture and the cell samples were incubated further at 37 °C for 4 h. Absorbance was measured at 450 nm by a Tecan P-200 fluorescence plate reader. Cell viability was expressed as a percentage of the absorbance in wells containing cells treated with amyloids compared to the control untreated cells.

### Graph analysis

In all TBI patient’s tissues the amounts of immunopositive plaques and cells reactive with each of studied antibodies were counted manually in the hippocampi and surrounding area. In order to reveal pair-wise relations between corresponding counts of immuno-positive cells and/or plaques, we subjected all paired data subsets to the graph theory analysis and calculations of Spearman’s rho correlations^67^. The nodes in graphs represent the type of immunopositive cells or plaques, which are connected by edges. The edges were drawn based on the moderate and strong Spearman’s rho correlations indicated along them (Supplementary Table S3).

### Fluorescence immunocytochemistry

Wild-type mouse primary neurons non-treated and treated with 1 µM Aβ_42_ oligomers for 24 h were imaged by a Leica Microsystems TCS SP8 confocal microscope equipped with a HP PL APO 63×/NA1.2 objective and using Diode 405/405 nm and Argon (405, 488, 552, 638 nm) lasers. Cell nulcei were stained with 4′,6-Diamidino-2-Phenylindole (DAPI), shown by blue fluorescence; Aβ_42_ oligomers were detected by using Aβ specific primary antibody and DyLight 488 secondary antibodies, shown by green fluorescence, while S100A9 was recognized by S100A9 specific primary antibodies and visialised by DyLight 594 secondary antibodies (Supplementary Table S2).

S100A9 specific immunofluorescence signal per cell in the Aβ_42_ treated and non-treated neurons were quantified by using an Imaris (Bitplane) software. For this purpose two-dimensional images obtained by confocal microscopy were reconstructed by Imaris into three-dimensional volumetric data sets with an inclusive volume for each neuronal cell set at 16 × 16 × 3 μm^3^ as shown in Supplementary Fig. S7. Using the same threshold level for treated and control neurons, solid iso-surfaces were defined for red channel (S100A9) and rendered as volumes, which were used for S100A9-specific immunofluorescence quantification presented in arbitrary units per cell. Results are presented as the mean ± standard deviation.

### Statistical analysis

The normality of the data sets was assessed by the Shapiro-Wilk test and the data were analyzed using Student’s *t* test. *p* value less than 0.05 was considered significant. Results are presented as the mean ± standard deviation.

## Electronic supplementary material

Supplementary Information
