# Supplementary material for: S100A9-Driven Amyloid-Neuroinflammatory Cascade in Traumatic Brain Injury as a Precursor State for Alzheimer’s Disease
Source: Sci Rep. 2018 Aug 27;8:12836. doi: 10.1038/s41598-018-31141-x (PMC6110751; doi:10.1038/s41598-018-31141-x)
Supplement: Supplementary file 1 — Supplementary Information [file 41598_2018_31141_MOESM1_ESM.pdf]

Supplementary Information

S100A9-Driven Amyloid-Neuroinflammatory Cascade in Traumatic Brain Injury as a Precursor State for Alzheimer's Disease

Chao Wang[1,§], Igor A. Iashchishyn[1,2,§], Jonathan Pansieri[1], Sofie Nyström[3], Oxana Klementieva[4], John Kara[1], Istvan Horvath[1], Roman Moskalenko[1,5], Reza Rofougaran[1], Gunnar Gouras[4], Gabor G. Kovacs[6], S.K. Shankar[7], Ludmilla A. Morozova-Roche[1]

[1]Department of Medical Biochemistry and Biophysics, Umeå University, 90187 Umeå, Sweden

[2]Department of General Chemistry, Sumy State University, Sumy 40000, Ukraine

[3]IFM-Department of Chemistry, Linköping University, Linköping, Sweden

[4]Department of Experimental Medical Sciences, Lund University, Lund, Sweden

[5]Department of Pathology, Sumy State University, Sumy 40000, Ukraine

[6]Institute of Neurology, Medical University of Vienna, Vienna, Austria

[7]Human Brain Tissue Repository, Department of Neuropathology, National Institute of Mental Health and Neurosciences, 560029 Bangalore, India

[§]First authors with equal contribution

Correspondence and request for materials should be addressed to C.W. (email: chao.wang@wustl.edu) or L.A.M-R (email: Ludmilla.Morozova-Roche@umu.se)

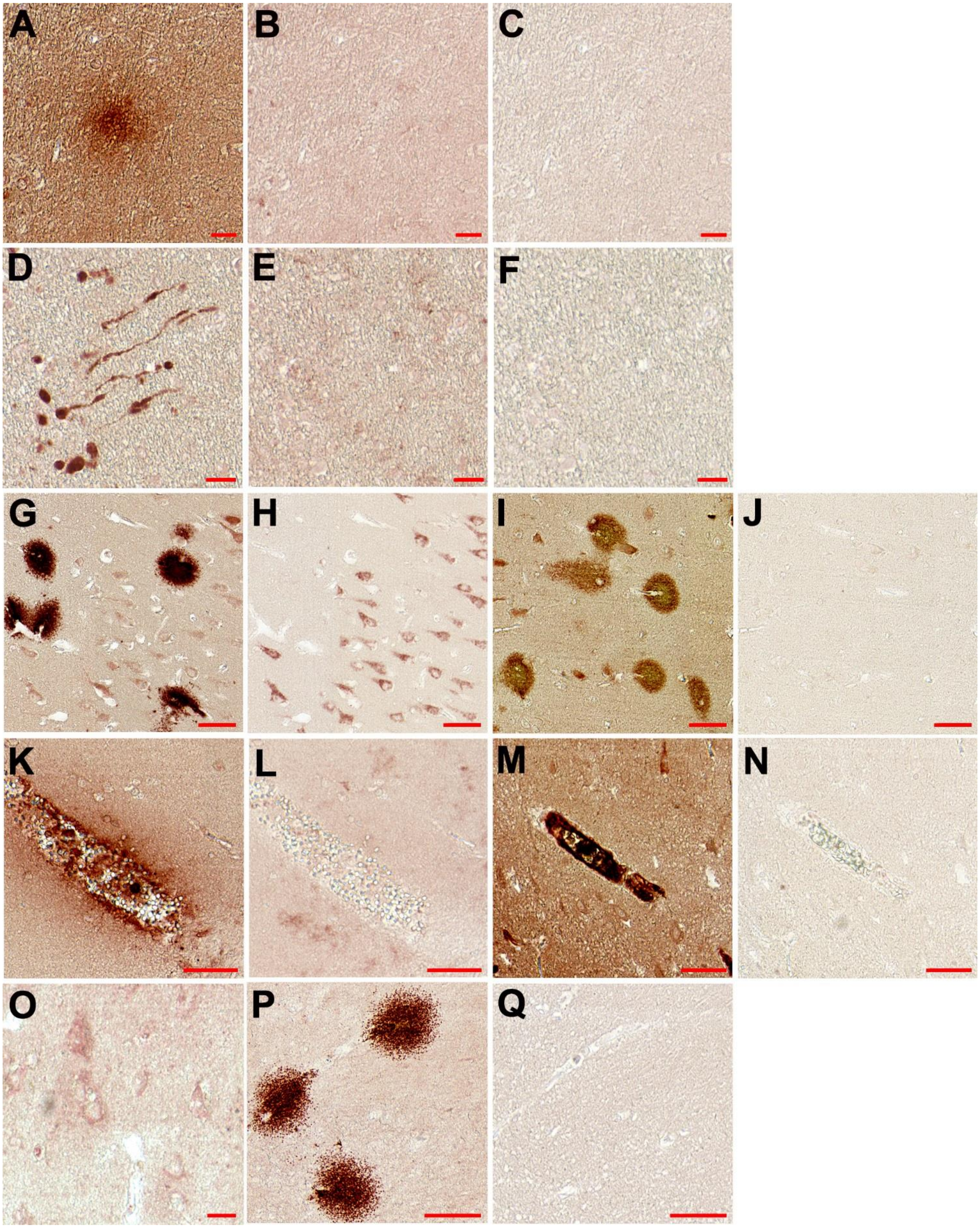
A
B
C
D
E
F
G
H
I
J
K
L
M
N
O
P
Q

Supplementary Fig. S1. S100A9 and Aβ in Human TBI Hippocampi. Sequential immunohistochemistry of a representative proteinaceous precursor-plaque with (A) Aβ, (B) amyloid oligomer specific A11 and (C) fibril specific OC antibodies, respectively (ca. 1 day post-TBI time). Sequential immunohistochemistry of neuronal cells with (D) Aβ, (E) amyloid oligomer specific A11 and (F) fibril specific OC antibodies, respectively (ca. 1 day post-TBI time). Pair of sequential immunohistochemistry images with (G) S100A9 and (H) A11 antibodies of TBI tissues (ca. 1 day post-TBI time) indicating that the precursor-plaques were reactive with S100A9 but not with A11 antibodies, while S100A9 immunopositive cells were stained also with A11 antibodies. Pair of sequential immunohistochemistry images with (I) S100A9 and (J) fibril specific OC antibodies of TBI tissues (ca. 1 day post-TBI time), indicating that the S100A9 immunopositive precursor-plaques were not reactive with OC antibodies. Pairs of sequential immunohistochemistry images of blood vessel in TBI tissues (ca. 1 day post-TBI time) with (K) S100A9−(L) A11 antibodies and (M) S100A9−(N) fibril specific OC antibodies, respectively. (O) Part of sequential immunohistochemistry procedure with S100A9 antibodies complementary to the image of Aβ immunopositive precursor-plaques in Figure 1B, indicating the lack of immunoreactivity of these plaques with S100A9 antibodies. Pair of sequential immunohistochemistry images of precursor-plaques in TBI brain tissues (ca. 1 day post-TBI) with (P) S100A9 and (Q) Aβ antibodies, respectively, indicating that the S100A9 positive precursor-plaques were not reactive with Aβ antibodies. Scale bars are 20 μm (A–F and O) and 50 μm in (G–N, P and Q).

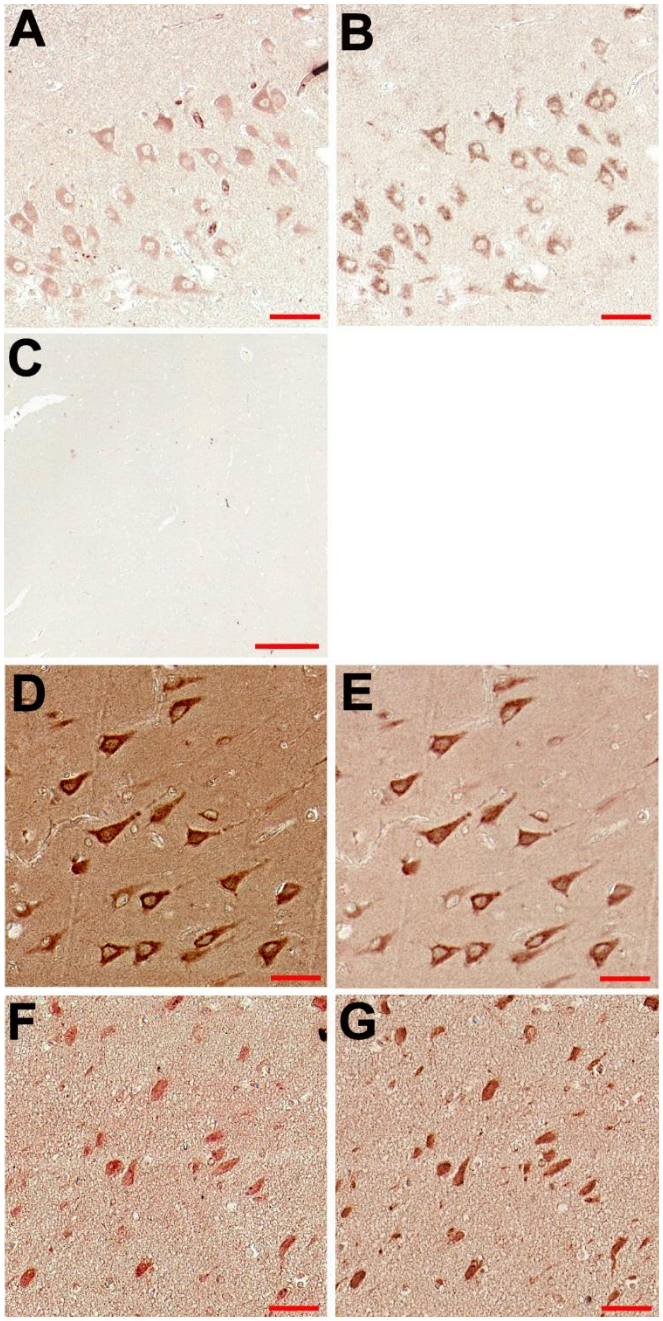

Supplementary Fig. S2. Intracellular S100A9/Aβ aggregation and apoptosis in human TBI hippocampi. Parts of sequential immunohistochemistry of neuronal cells in the TBI hippocampus with (A) S100A9 and (B) A11 antibodies, respectively, which are complementary to the Aβ-positive immunostaining (Figure 2K) (C) Representative image of double immunohistochemistry with S100A9 and Aβ antibodies of the hippocampus tissues in control non-demented patient, showing lack of immunostaining. Parts of sequential immunohistochemistry of neuronal cells in the TBI hippocampus with (D) S100A9 and (E) A11 antibodies,

respectively, which are complementary to the Bax-positive immunostaining (Figure 2L). (F and G) Parts of sequential immunohistochemistry of neuronal cells with (F) S100A9 and (G) A11 antibodies, respectively, which are complementary to the immunostaining with activated caspase-3 antibodies (Figure 2M). Scale bars are 50 µm (A, B, D–G) and 500 µm (C).

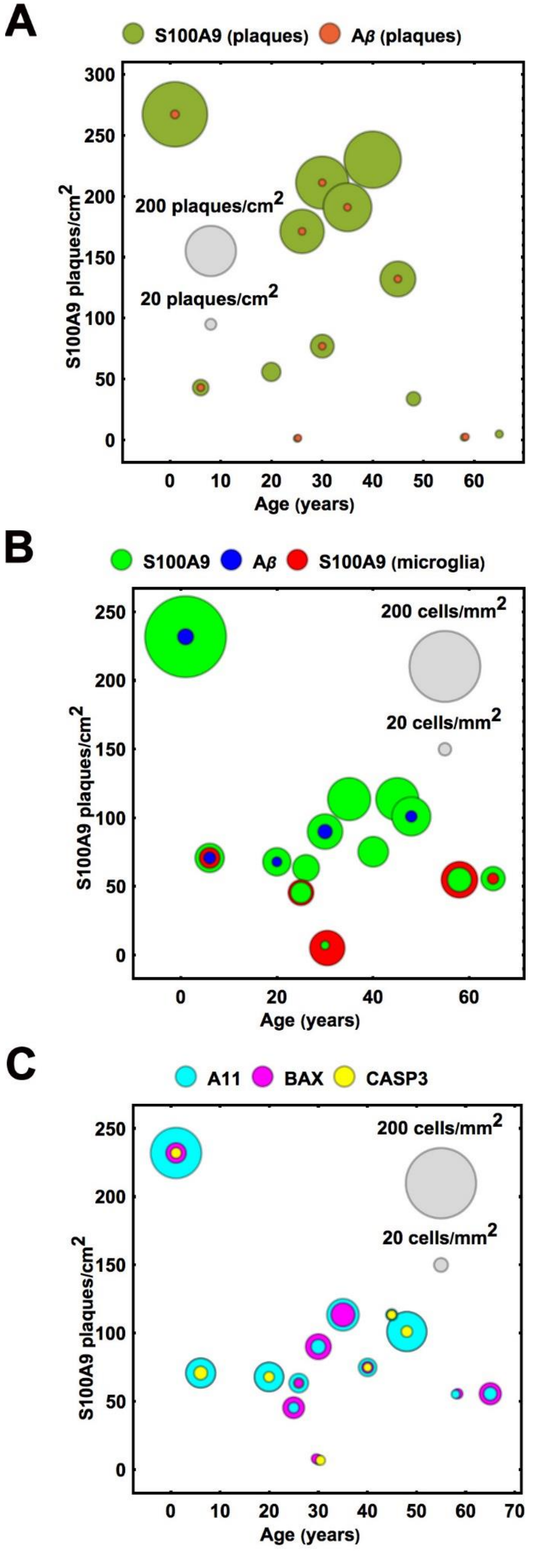
A
S100A9 (plaques)
Aβ (plaques)
S100A9 plaques/cm2
200 plaques/cm2
20 plaques/cm2
Age (years)
B
S100A9
Aβ
S100A9 (microglia)
S100A9 plaques/cm2
200 cells/mm2
20 cells/mm2
Age (years)
C
A11
BAX
CASP3
S100A9 plaques/cm2
200 cells/mm2
20 cells/mm2
Age (years)

Supplementary Fig. S3. Effect of age on TBI amyloid, neuroinflammatory and apoptotic responses. n=13 TBI patients. Bubble charts showing counts of (A) the precursor-plaques reactive with S100A9 and Aβ antibodies, (B) neuronal and microglial cells reactive with S100A9 and Aβ antibodies and (C) neuronal cells reactive with A11, Bax and activated caspase-3 antibodies, respectively, depending on the subject age. Each bubble corresponds to individual subject.

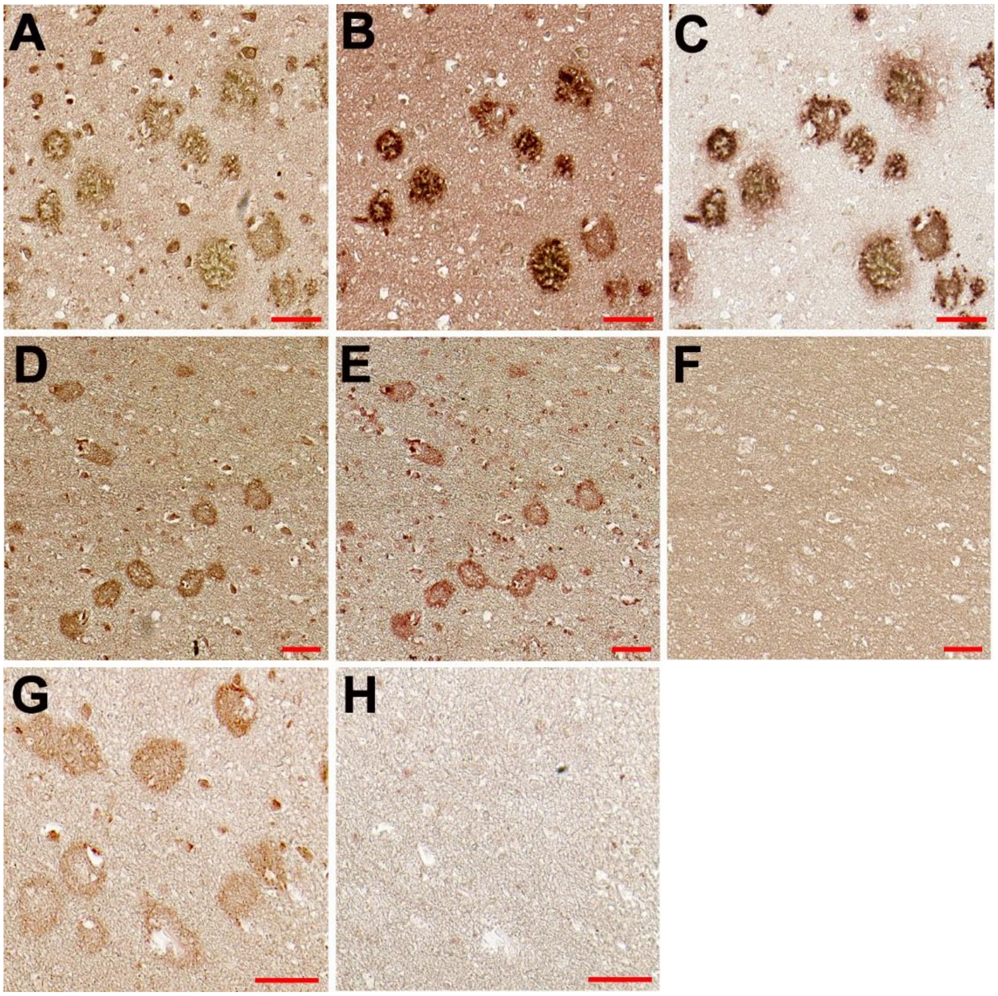

Supplementary Fig. S4. S100A9 and Aβ plaques in human brain tissues with Alzheimer's disease. Senile plaques stained with sequence of (A) S100A9, (B) Aβ and (C) OC antibodies. S100A9 plaques stained with sequence of (D) S100A9, (E) A11 and (F) Aβ antibodies. S100A9 plaques stained with sequence of (G) S100A9 and (H) OC antibodies. Scale bars are 50 μm in (A–H).

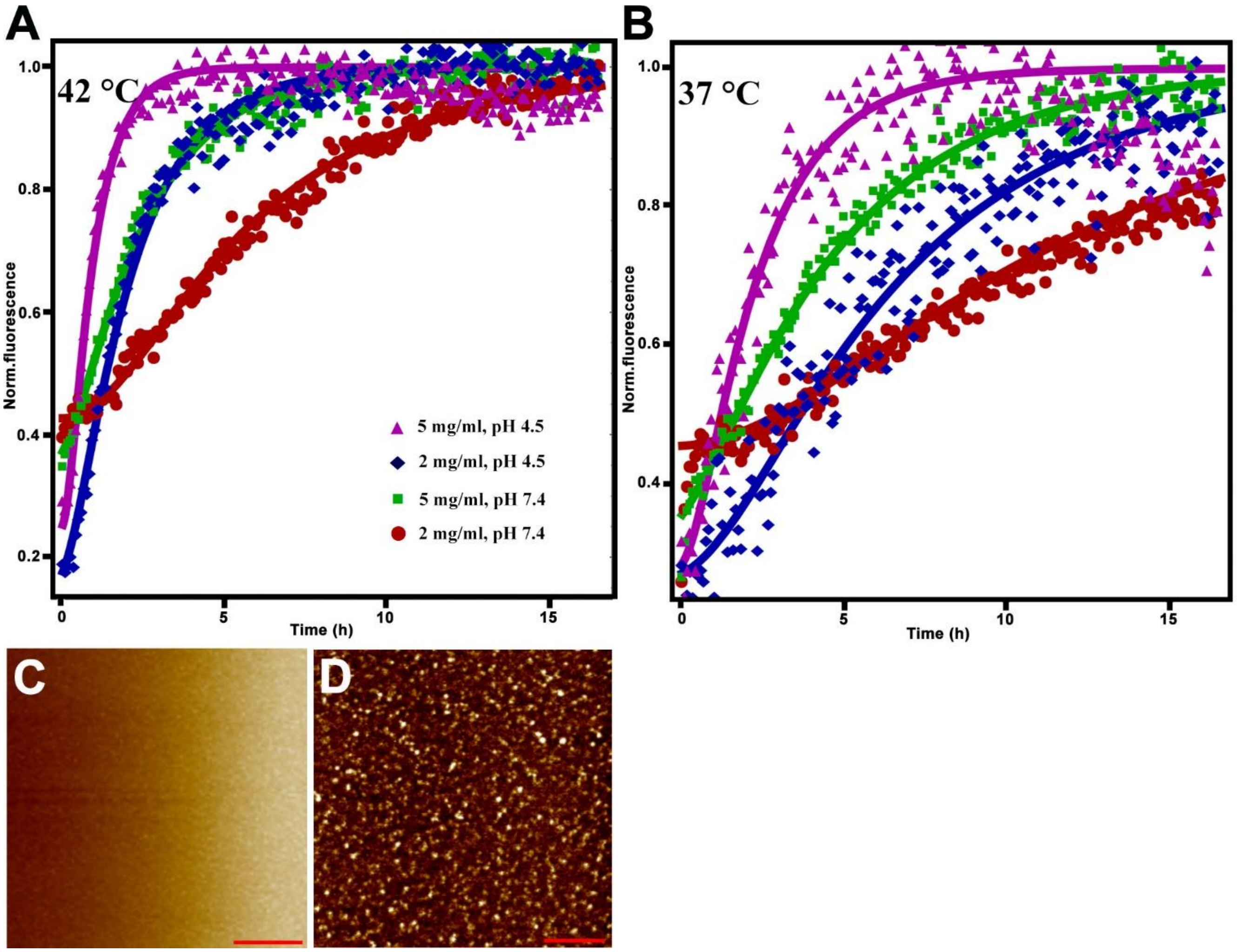

Supplementary Fig. S5. *In vitro* S100A9 amyloid formation and proteinase K digestion. (A and B) Normalized kinetics of S100A9 amyloid formation monitored by h-FTAA fluorescence at (A) 42 °C and (B) 37 °C under shaking with glass beads. S100A9 amyloid kinetics in 10 mM PBS, pH 7.4, 2 mg/ml is shown in red and at 5 mg/ml – in green; amyloid kinetics in 20 mM sodium acetate, pH 4.5, 2 mg/ml is shown in blue and at 5 mg/ml – in magenta. (C) AFM height images of S100A9 amyloids (5 mg/ml) subjected to 72 h treatment with proteinase K at pH 7.4, 37 °C and (D) – at pH 4.5, 42 °C. Scale bars are 2 µm in (C) and 200 nm in (D).

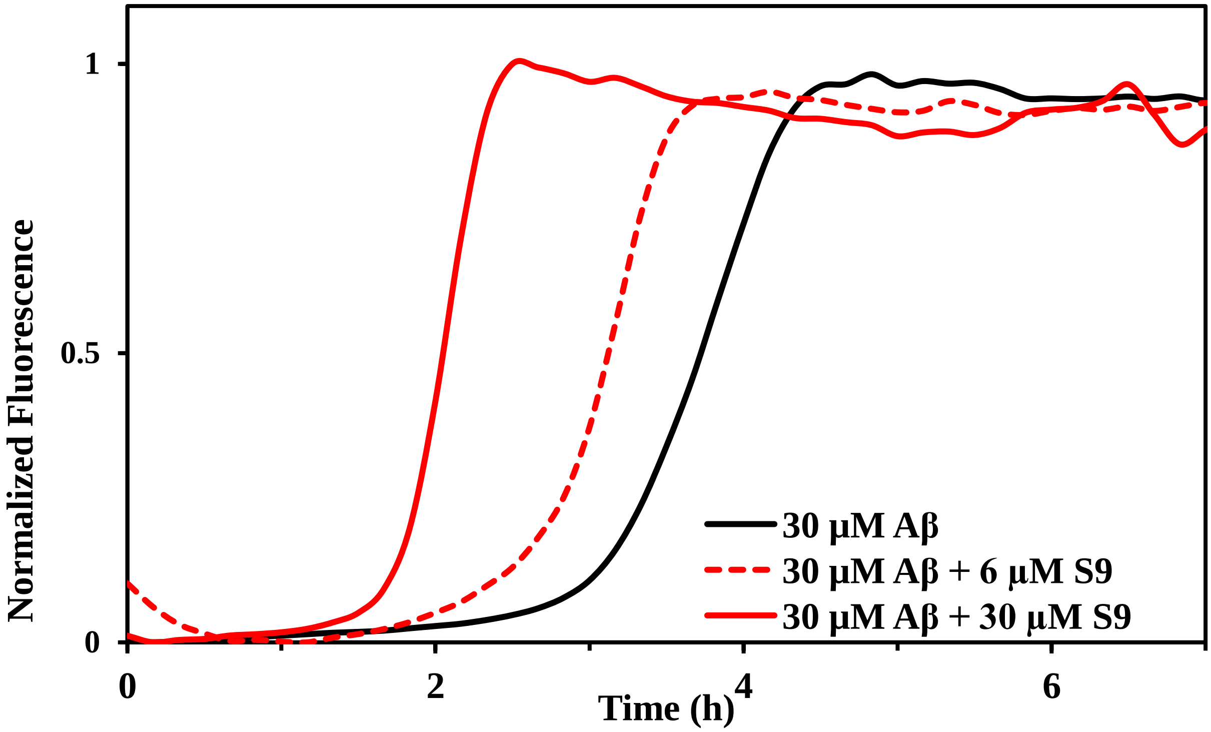

Supplementary Fig. S6. Effect of S100A9 fibrillar sample on Aβ_{42} amyloid formation kinetics monitored by thioflavin-T fluorescence assay. Concentrations of Aβ_{42} and S100A9 samples are indicated in caption.

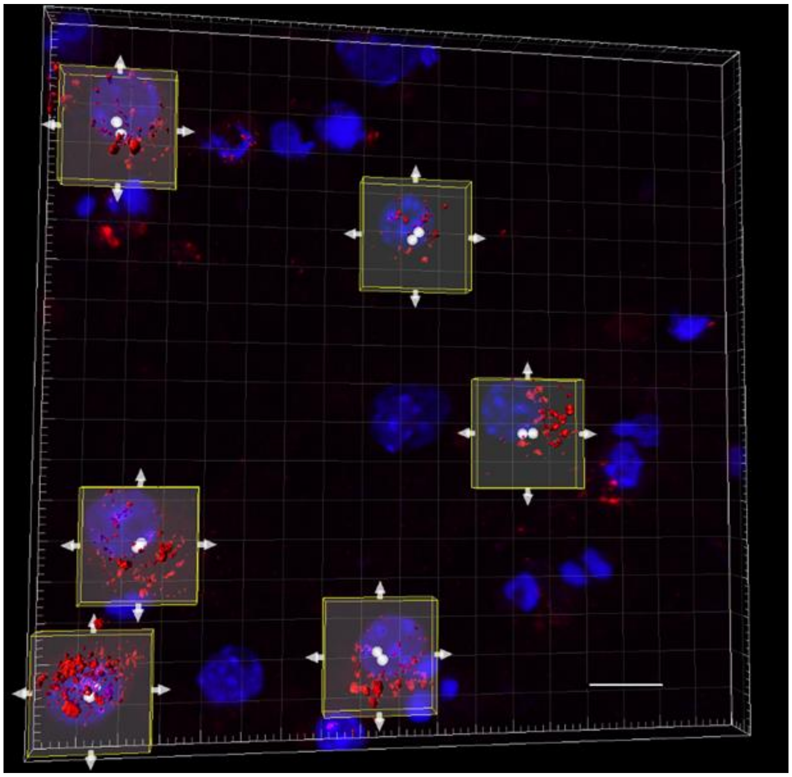

Supplementary Fig. S7. Representative confocal microscopy image of wild-type mouse neurons expressing S100A9 (shown by red immunofluorescence) induced by addition of Aβ_{42} oligomers. DAPI nuclei staining shown in blue. S100A9-specific immunofluorescence signal per cell was quantified by using an Imaris (Bitplane) software. Two-dimensional cell images obtained by confocal microscopy were reconstructed by an Imaris into three-dimensional volumetric data sets and the volumetric data, reflecting S100A9, rendered iso-surfaces used for quantification. Scale bar is 10 µm.

Supplementary Table S1. Characteristics of TBI, SMCI, AD and control patients.

	Case number	Age	Sex	Diagnosis	Survival time	Objects
TBI	1	48	M	RTA TBI	4 h	Hippocampus
	2	45	M	RTA TBI	6 h	Hippocampus
	3	30	M	RTA TBI	ca. 0.5 day	Hippocampus
	4	40	M	RTA TBI	ca. 0.5 day	Hippocampus
	5	20	M	RTA TBI	ca. 1 day	Hippocampus
	6	1	F	RTA TBI	ca. 1 day	Hippocampus
	7	26	M	RTA TBI	ca. 1 day	Hippocampus
	8	35	M	RTA TBI	ca. 2 day	Hippocampus
	9	6	F	RTA TBI	4.5 days	Hippocampus
	10	65	F	RTA TBI	5 days	Hippocampus
	11	30	M	RTA TBI	9 days	Hippocampus
	12	25	M	RTA TBI	10 days	Hippocampus
	13	58	M	RTA TBI	1 month	Hippocampus
SMCI	1	43	M	SMCI; cirrhosis of liver; 5 years military service		Frontal lobe
AD	1	57	M	AD, Braak stage VI; Motor vehicle accident in 2002 and died in 2012		Temporal lobe
	2	69	M	AD, Braak stage VI		Temporal lobe
	3	87	M	AD, Braak stage IV		Temporal lobe
	4	88	F	AD, Braak stage VI		Temporal lobe
	5	86	F	AD, Braak stage VI		Temporal lobe
	6	83	M	AD, Braak stage VI		Frontal lobe
Control	1	35	M	RTA – Skull fracture and the brain came out		Hippocampus
	2	30	F	Acute leucosis		Hippocampus
	3	75	M	COPD		Hippocampus

RTA = road traffic accident; COPD = Chronic obstructive pulmonary disease.

Supplementary Table S2. Reagents and antibodies for immunohistochemistry and immunocytochemistry.

	Product name	Dilution	Catalogue number	Supplier	Epitope
Primary antibody	Rabbit anti-S100A9	1/100	sc-20173	Santa Cruz Biotechnology	Polyclonal, human S100A9 mapping to 1q21.3
	Goat anti-S100A9	1/100	sc-8115	Santa Cruz Biotechnology	Polyclonal, mouse S100A9 mapping to 3 F1
	Mouse anti-S100A8	1/50	MAB4570	R&D Systems	Monoclonal, human S100A8 mapping to aa 1-93
	Rabbit anti-A11	1/500	–	Generated by R. Kayed	Generic amyloid oligomers
	Rabbit anti-OC	1/1000	–	Generated by R. Kayed	Generic amyloid firbils
	Mouse anti-Aβ	1/100	ab11132	Abcam	Monoclonal, human Aβ aa 1-17
	Mouse 6E10 anti-Aβ	1/100	SIG-39320	Biolegend	Monoclonal, human Aβ aa 3-8
	Mouse anti-CD68	1/100	sc-70761	Santa Cruz Biotechnology	Monoclonal, human CD68 mapping to 17p13.1
	Rabbit anti-NeuN	1/500	ab104225	Abcam	Polyclonal, human NeuN mapping to aa 1-100
	Mouse anti-Bax	1/100	sc-7480	Santa Cruz Biotechnology	Monoclonal, mouse Bax mapping to aa 1-171
	Rabbit anti-active caspase-3	1/50	ab2302	Abcam	Polyclonal, human activated caspase-3
Secondary antibody	Anti-rabbit	–	MP-7401	Vector Laboratories	–
	Anti-mouse	–	MP-7402	Vector Laboratories	–
	Anti-goat	1/200	ab6885	Abcam	–
	DyLight 488 anti-mouse IgG	1/100	35502	Thermo Fisher Scientific	–
	DyLight 594 anti-mouse IgG	1/100	35510	Thermo Fisher Scientific	–
	AEC peroxidase substrate		SK-4205	Vector Laboratories	–
	AQ aqueous mounting medium		H-5501	Vector Laboratories	–

Supplementary Table S3. Spearman's rho correlations between immunopositive cell and plaque counts.

Spearman's Rho

	S100A9 (plaques)	Aβ (plaques)	S100A9	Aβ	S100A9 (microglia)	A11	BAX	CASP3
S100A9 (plaques)	1.	0.39	0.65	0.29	-0.69	0.45	-0.2	0.14
Aβ (plaques)	0.39	1.	0.3	0.24	0.	0.15	-0.35	0.32
S100A9	0.65	0.3	1.	0.49	-0.78	0.73	0.26	0.25
Aβ	0.29	0.24	0.49	1.	-0.32	0.62	0.61	0.54
S100A9 (microglia)	-0.69	0.	-0.78	-0.32	1.	-0.67	-0.15	-0.24
A11	0.45	0.15	0.73	0.62	-0.67	1.	0.53	0.6
BAX	-0.2	-0.35	0.26	0.61	-0.15	0.53	1.	0.24
CASP3	0.14	0.32	0.25	0.54	-0.24	0.6	0.24	1.

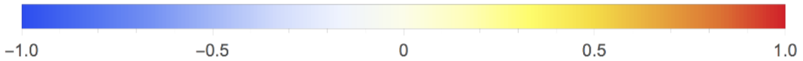

Recalculation of the correlation coefficients for the group of TBI patients without two young individuals does not affect significantly the values of these coefficients**.**

Supplementary Methods

Preparation of S100A9 amyloid fibrils

Freeze-dried S100A9 protein was dissolved in PBS buffer at pH 7.4. The solution was filtered through a spin 0.2 μm membrane filter to remove any aggregated species. Fibrils were prepared by incubating S100A9 solution (final concentration of 400 μM) in PBS buffer, pH 7.4, at 50 °C in 500 μl reaction volume on a rotating shaker (300 rpm) during 3 days. Fibril morphology was confirmed by AFM.

Thioflavin-T kinetics assays

$A\beta_{42}$ (Tocris Bioscience) was freshly dissolved and filtered through a spin 0.2 μm membrane filter to remove aggregated species. 30 μM $A\beta_{42}$ in PBS buffer, pH 7.4, was incubated alone and in the presence of S100A9 fibrillar samples taken at 6 and 30 μM concentrations in a 96-well plate mixed with 20 μM thioflavin-T at 42°C. The resulting fluorescence was measured each 10 minutes during 13 hours using a fluorescence plate reader (Infinite F200 pro, TECAN) in triplicate samples. Excitation and emission wavelengths were set at 430 nm and 485 nm, respectively.
